# Neurokinin1 − cholinergic receptor mechanisms in the medial Septum-Dorsal hippocampus axis mediates experimental neuropathic pain

**DOI:** 10.1016/j.ynpai.2024.100162

**Published:** 2024-08-03

**Authors:** Mohammed Zacky Ariffin, Si Yun Ng, Hamzah Nadia, Darrel Koh, Natasha Loh, Naomi Michiko, Sanjay Khanna

**Affiliations:** a Department of Physiology, Yong Loo Lin School of Medicine, National University of Singapore, Singapore; b Neurobiology Programme, Life Sciences Institute, National University of Singapore, Singapore; c Healthy Longevity Translational Research Programme, Yong Loo Lin School of Medicine, National University of Singapore, Singapore

**Keywords:** Medial septum, Cholinergic neurons, Neurokinin transmission, Nociception, Chronic constriction injury

## Abstract

The neurokinin-1 receptors (NK1Rs) in the forebrain medial septum (MS) region are localized exclusively on cholinergic neurons that partly project to the hippocampus and the cingulate cortex (Cg), regions implicated in nociception. In the present study, we explored the hypothesis that neurotransmission at septal NK1R and hippocampal cholinergic mechanisms mediate experimental neuropathic pain in the rodent chronic constriction injury model (CCI). Our investigations showed that intraseptal microinjection of substance P (SP) in rat evoked a peripheral hypersensitivity (PH)-like response in uninjured animals that was attenuated by systemic atropine sulphate, a muscarinic-cholinergic receptor antagonist. Conversely, pre-emptive destruction of septal cholinergic neurons attenuated the development of PH in the CCI model that also prevented the expression of cellular markers of nociception in the spinal cord and the forebrain. Likewise, anti-nociception was evoked on intraseptal microinjection of L-733,060, an antagonist at NK1Rs, and on bilateral or unilateral microinjection of the cholinergic receptor antagonists, atropine or mecamylamine, into the different regions of the dorsal hippocampus (dH) or on bilateral microinjection into the Cg. Interestingly, the effect of L-733,060 was accompanied with a widespread decreased in levels of CCI-induced nociceptive cellular markers in forebrain that was not secondary to behaviour, suggesting an active modulation of nociceptive processing by transmission at NK1R in the medial septum. The preceding suggest that the development and maintenance of neuropathic nociception is facilitated by septal NK1R-dH cholinergic mechanisms which co-ordinately affect nociceptive processing in the dH and the Cg. Additionally, the data points to a potential strategy for pain modulation that combines anticholinergics and anti-NKRs.

## Highlights

• Excitation of septal cholinergic neurons mimicked nociception.
• Cholinergic loss in septo-dorsal hippocampus elicited antinociception.
• NK1R antagonist reduce septal cholinergic function and is antinociceptive.
• NK1R-cholinergic axis in septo-dorsal hippocampus is pro-nociceptive.

## Introduction

The medial septum (MS) region is implicated in pain in humans (Keifer et al., 2014) and nociception in animals (Ang et al., 2017, Lee et al., 2011, Takeuchi et al., 2021). A combination of findings suggests that the septal cholinergic neurons facilitate nociception. Accordingly, the septal cholinergic neurons project to both the hippocampus and the medial prefrontal cortex, regions implicated in pain (Amaral and Kurz, 1985, Baliki and Apkarian, 2015, Khanna and Sinclair, 1989, Senut et al., 1989). Further, c-Fos, an immediate early gene that is a marker of neuronal activation, is induced in septal cholinergic neurons in the complete Freund’s adjuvant model of persistent inflammatory pain, while, conversely, chemogenetic inactivation of septo-cingulate cholinergic neurons is antinociceptive in the model (Jiang et al., 2018).

Additionally, hind paw injection of the noxious agent, formalin, induces an increase in acetylcholine release in hippocampus that receives cholinergic input exclusively from the MS region (Aloisi et al., 1997). Conversely, the destruction of septal cholinergic neurons or iontophoresis of cholinergic muscarinic receptor antagonist, atropine, into dorsal hippocampus (dH) CA1 attenuates the formalin-induced suppression of CA1 pyramidal cell excitability (Khanna and Sinclair, 1992, Zheng and Khanna, 2001). However, the role of cholinergic transmission in dH in mediating persistent nociception is not known.

Interestingly, the septal cholinergic neurons exclusively express neurokinin 1 receptors (NK1Rs) in the MS region (Chen et al., 2001, Steinberg et al., 1998). The NK1Rs are canonical receptor for substance P (SP) and are implicated in mediation of nociception at the spinal and brainstem levels (Hamity et al., 2010, Jensen et al., 2017; Zieglgänsberger, 2019). More recently, the NK1R in the MS region have been implicated in acute nociception such that intraseptal microinjection of L-733,060, an antagonist at NK1Rs, attenuated both the neural responses in dH field CA1 and nociceptive behaviour in the formalin model (Ng et al., 2021). Indeed, neurochemical findings raise a possibility of a functional role of forebrain neurokinin-1 receptors (NK1Rs) in chronic pain in humans and animals. For example, the availability of NK1R in human and the expression level of NK1R mRNA in rodent is decreased in forebrain regions, including the hippocampus, in clinical and experimental pain, respectively (Duric and McCarson, 2005, Duric and McCarson, 2007, Jarcho et al., 2013, Linnman et al., 2010). Further, SP levels are increased in CSF of patients with fibromyalgia syndrome, a chronic pain disorder (Russell et al., 1994, Vaerøy et al., 1988). Nonetheless, whether the forebrain NK1R, especially in the MS region, mediate persistent nociception remains unclear.

Given the above background, we hypothesized that the septal NK1R mechanism in the MS, responding to nociceptive inputs, and dH cholinergic mechanisms, reflecting MS output, mediate neuropathic nociception. To test this hypothesis, we have examined whether: (a) intraseptal SP modulates nociception in a cholinergic receptor-dependent mechanism, (b) pre-emptive destruction of septal cholinergic neurons affect nociception, and (c) intraseptal L-733,060 or intrahippocampal cholinergic receptor antagonists modulate nociception in the rodent chronic constriction injury (CCI) model of neuropathic pain. Arising from our hypothesis, we reasoned that each of the above manipulations will be antinociceptive. Also, while both dH and ventral hippocampus (vH) modulate nociception, we have focussed on septo-dH for the following reasons: (a) manipulation of dH can affect nociception independently of vH (Wei et al., 2021), (b) dH and vH are innervated by separate populations of septal cholinergic neurons that serve different functions (Li et al., 2022), and (c) stimulation of septo-vH cholinergic neurons is antinociceptive, while, conversely, we are testing whether activation of septal NKR-dH cholinergic axis is pro-nociceptive. The current hypothesis follows our previous investigation as referred to in the text above.

## Material and methods

### 2.1 Animals

All animal experiments have complied with the ARRIVE Guidelines. Pharmacological investigations were carried out on male Sprague-Dawley rats (SD; 300–350 g at the start of experiments: InVivos Pte Ltd, Singapore). Male animals were used to remain consistent with previously collected data (Ariffin et al., 2018, Ng et al., 2021).

All experiments followed the Ethical Guidelines of the International Association for the Study of Pain. Experimental procedures were approved by the Institutional Animal Care and Use Committee (IACUC) at the National University of Singapore, with efforts taken to minimize the number of animals used.

Animals were randomly assigned to each experimental group. The researchers were blind to the treatment administered to the animals.

### 2.2 Drugs and drug dosage

The following drugs were microinjected into the MS region: (a) Substance P (SP; 2 or 7 µg/µl, 0.5 µl; #S6883, Sigma, USA), an NK1R agonist, (b) L-733,060 (0.0176 µg/µl, 0.5 µl; #1145, Tocris Bioscience, UK), an NK1R antagonist (Hamity et al., 2010), (c) carbamylcholine chloride (carbachol; 0.156 µg/µl, 0.5 µl; C4382, Sigma, USA), a broad-spectrum cholinergic agonist, and (d) 192 IgG-Saporin (192 IgG-SAP; 0.42 µg/µl, total volume of 2 µl; IT-01, Advanced Targeting Systems, USA), an immunotoxin that selectively destroys septal cholinergic neurons.

The dose of L-733,060, carbachol and 192 IgG-SAP was based on published findings from the laboratory (Ariffin et al., 2013, Ariffin et al., 2017, Jiang and Khanna, 2006, Ng et al., 2021, Zheng and Khanna, 2001). In context of SP, a comparison was made between different doses based, on which the SP concentration of 2 µg/µl was used in most experiments (see ‘2.6 Selection of SP concentration’*)*.

The following drugs were microinjected into the dorsal hippocampus (dH) or the medial prefrontal cortex (mPFC): (a) mecamylamine hydrochloride, (2 µg/µl; M9020, Sigma, USA; 0.5 µl/site), a broad-spectrum antagonist at nicotinic cholinergic receptors and (b) atropine sulphate (0.007 µg/µl; A0257, Sigma, USA; 0.5 µl/site), a broad-spectrum antagonist at muscarinic cholinergic receptors. The concentration of the drugs used was based on published data from the laboratory (Ariffin et al., 2010).

The locally administered drugs were dissolved in the vehicle made of 0.5 % w/v Alcian blue dye solution in saline. As control, vehicle was microinjected into the region of interest.

In some animals, atropine sulphate (5 mg/kg, i.p.; A0257, Sigma, USA) was administered systematically to examine the involvement of cholinergic neurotransmission in mediating observed effects. In this instance, atropine was dissolved in saline.

### 2.3 Drug microinjection

In the anaesthetised animals, drug microinjections were performed using a 33G microinjection needle attached to a microsyringe (Ito Corporation, Japan). The needle was lowered into the septal region using stereotaxic technique. The drug was microinjected over a period of 30 s.

Drug microinjections in awake animals were performed by gently restraining the animal and inserting a 33G internal cannula (PlasticsOne, USA) into a guide cannula that was previously implanted (see below). The internal cannula protruded by 1.0 mm from the tip of the implanted guide cannula. The internal cannula was attached to a microsyringe (Ito Corporation, Japan) via polythene tubing. The drug was microinjected over a period of 30 s. The internal cannula was left in place for 1 min after the drug administration to facilitate drug diffusion and to prevent backflow into the guide cannula.

### 2.4 Experimental preparations

*Anaesthetized animals-preparation for electrophysiological recording:* Experiments were performed in anaesthetized SD rats to investigate the effects of intraseptal microinjection of selected drugs on hippocampal neural responses. All animals were anaesthetized with urethane (1 g/kg, i.p.; Sigma, USA) and mounted onto a stereotaxic frame. Body temperature was maintained with a thermal blanket (Harvard Apparatus, USA).

Extracellular field potentials were recorded from the left hippocampal field CA1 (P3.6 mm from Bregma, L2.4 mm from midline, and V4.0 mm from the cortical surface) (Paxinos and Watson, 2013). A saline filled carbon fibre glass electrode was lowered at 10 degrees to left of the vertical towards the left hippocampal field CA1. Signal recorded by the carbon fibre glass electrode was used to record the CA1 population spike (PS; amplified 10,000X with Grass amplifier from Astromed USA, band pass filtered at 1–3000 Hz and digitized at 10 kHz using Power 1401 and Spike2 software from Cambridge Electronic Design or CED, UK) from the pyramidal cell layer. The PS was evoked by stimulating (0.01 s pulse duration at 0.1 Hz) a concentric bipolar electrode (Model NE-100, David Kopf, USA) through a constant current stimulation isolation unit (Grass S88 stimulator; Grass Technologies, USA). The electrode was positioned in the left hippocampal CA3 region (P3.0 mm from Bregma, L2.4 mm from midline, and V4.0 mm from the cortical surface) (Paxinos and Watson, 2013). CA3 stimulation intensity was adjusted to generate a PS amplitude of 70 % of the maximum under large irregular field activity.

*Survival surgery – implanting microinjection cannula, microinjection of 192 IgG-SAP*: Stereotactic surgery was performed on rats under aseptic conditions. Anaesthesia was induced and maintained with 5 % and 2 % isoflurane, respectively, with oxygen flow at 1 L/min. All implants were secured with dental cement and support screws.

Rats were implanted with microinjection cannula for drug administration. A single barrel 26G stainless steel guide cannula (Plastic One, USA) was implanted either into the MS (A0.5 mm from Bregma, L0.0 mm, V5.5 mm from the cortical surface) (Paxinos and Watson, 2013), or unilaterally into dH (P3.3 mm from Bregma, L±2.5 mm from midline, V2.5–3.0 mm from skull surface). In other instances, the guide cannulas were implanted bilaterally into dH, or into the mPFC (A2.0 mm from Bregma, L±1.1 mm from midline, V2.1 mm from skull surface) (Paxinos and Watson, 2013). Bilateral implants were angled 10 degrees to the vertical.

In another group of rats, the animals were microinjected with 192 IgG-SAP (0.42 µg/µl; Advanced Targeting Systems, USA) into the MS (A0.5 mm from Bregma, L±0.2 mm from midline, V7.0 mm and 5.5 mm from skull surface) with a microsyringe (Ariffin et al., 2013, Paxinos and Watson, 2013). The immunotoxin selectively destroys septal cholinergic neurons (Book et al., 1994, Ariffin et al., 2013, Zheng and Khanna, 2001). 0.5 µl of the 192 IgG-SAP or the corresponding vehicle was microinjected at each microinjection site over a period of 1 min. At each site, the needle was left in position for 5 min to facilitate diffusion and minimize back flow.

On recovery from anaesthesia, animals were singly housed and administered with post-operative analgesic Buprenorphine (0.05 mg/kg, i.p.; twice a day for 3 consecutive days) and antibiotic Enrofloxacin (10 mg/kg, i.p.; once a day for 5 consecutive days). Animals were allowed to recover before performing an additional surgery to ligate the sciatic nerve (see below). Animals that were microinjected with 192 IgG-SAP were ligated 17 days after surgery and microinjection. The remaining animals were allowed to recover for 7 days before initiating experiments.

The incisions made during surgeries were closed with sutures 3/0 nylon sutures (Dermalon; Covidien, UK).

*Chronic Constriction Injury model of neuropathic pain*: Chronic constriction injury (CCI), a model of neuropathic pain (Bennett and Xie, 1988), was induced as described previously (Ariffin et al., 2018). Briefly, the right sciatic nerve was exposed and isolated from surrounding perineural tissue. Three ligations of chromic catgut sutures (4/0; B.Braun, Germany), spaced 1 mm apart, were tied loosely around the right sciatic nerve. The muscle and skin layers were closed with absorbable sutures. Sham ligated animals underwent the same surgical procedure but without ligation. The animals were allowed to recover for at least 4 days before initiating experiments. However, note that neither buprenorphine nor enrofloxacin were administered following ligation or sham surgery.

### 2.5 Behavioural tests

*Mechanical and thermal stimulation:* As described previously (Ariffin et al., 2018), the paw withdrawal threshold (PWT) to mechanical stimuli paw and the withdrawal latency (PWL) to thermal stimulation of the hind paw was measured using an anesthesiometer (Ugo Basile, Italy) and the plantar test apparatus (Ugo Basile, Italy), respectively. The PWT and PWL were measured on alternate days.

Mechanical stimulus (20–50 g in 5 g increments) was applied to the plantar surface of the hind paws via a steel filament, while noxious thermal stimulus was applied via a radiant heat source placed under the chamber and directed towards the plantar surface of the hind paws. A noxious thermal stimulus of 40 arbitrary units was applied to the hind paws of rats to generate the paw withdrawal. The mechanical and thermal stimuli were applied when the animal was in still alert position i.e., on all four paws, head held up, and not moving. The stimulus was applied with an inter paw-stimulus interval of at least 30 s. Thus, a stimulus was re-applied to a given paw at an interval of at least 60 s.

During measurement of PWT, each intensity of the mechanical stimulus was applied six times to a paw for a maximum duration of 6 s. A positive response was considered when the animal lifted or flinched the tested paw with a latency of less than 6 s. The PWT of a paw was the stimulus intensity that elicited at least four positive responses from six application of mechanical stimuli to the paw.

The noxious thermal stimulus was applied to the plantar surface of the hind paws for a maximum duration of 20 s. Generally, the selected thermal stimulus evoked a PWL of around 15 s in the animals. Stimulus was applied five times to each paw. The PWL of a paw was the average of the times taken to respond to five applications of thermal stimulus to a paw.

*Place conditioning*: As described previously (Ang et al., 2015, Ariffin et al., 2018), animal conditioned place aversion (CPA) or the conditioned place preference (CPP) in CCI-ligated animals, a surrogate measure of spontaneous nociception, were measured using a common apparatus that consisted of two chambers separated by a movable barrier (12 x 40 cm). CPA and CPP was performed on separate group of animals. The two chambers of the apparatus were distinguished by visual and tactile cues − one chamber had a smooth floor with white walls while the other chamber had a rough floor with black and white striped walls. The Actimot software (TSE, Germany) tracked the movements of the animal. The software also calculated the time spent by the animal in each chamber.

The experiments were carried out over four consecutive days (CPA1-4/CPP1-4). During pre-conditioning sessions (CPA1-2/CPP1-2), each session lasting 15 min, animals were free to explore the two chambers of the apparatus. The chamber in which the animal spent the most time during each exposure was designated as the preferred chamber. Animals were excluded from experiments if they showed i) a bias by spending more than 80 % of the total time in a single chamber, or ii) switched chamber preference between the two sessions.

During conditioning (CPA3/CPP3), animals were exposed to the two chambers of the apparatus in two separate sessions of 1 hr each. The sessions were 3 hr apart. The chambers were isolated during each session by the barrier. In the first session of the CPA3 and CPP3, the animal was placed in the non-preferred and preferred chamber, respectively, following intraseptal microinjection of vehicle. In the second session of the CPA3 and CPP3, the animal was placed in the chamber opposite to the first session following intraseptal microinjection of SP (SP, 2 µg/µl, 0.5 µl; CPA3) or the L733,060 (0.0176 µg/µl, 0.5 µl; CPP3). In control animals, a second microinjection of vehicle was administered into the septum. The drug or vehicle was microinjected 5 min before placing the animals in the chamber. The next day, i.e., the CPA4 and CPP4, the animal was re-exposed to the apparatus for 15 min without the barrier. The time spent in each of the two chambers was recorded.

### 2.6 Experimental protocol

*Selection of SP concentration (see ‘Results’)*: The electrophysiological effect of microinjection of a high concentration of SP (7 µg/µl, 0.5 µl) was examined on the amplitude of hippocampal field CA1 PS. The amplitude of the PS preceding microinjection was set at 70 % of maximal amplitude observed during period of large irregular field activity (LIA). The PS was monitored for 2 min before and 20 min after microinjection and again at 40th and 60th min post microinjection. The effect of the high dose of SP on amplitude of PS was compared with the previously reported responses to the lower concentrations of SP (1 µg/µl or 2 µg/µl; see ‘Results’) (Ng et al., 2021). Control animals were microinjected with equivalent volume of saline.

The SP-induced suppression of CA1 PS, but not theta wave field activity, was used as the electrophysiological readout since the agonist induces little or no theta activation (Ng et al., 2021).

Based on the comparison, the SP concentration of 2 µg/µl was used in all subsequent experiments performed with SP in the current study (see ‘Results’).

**Fig. 1:**
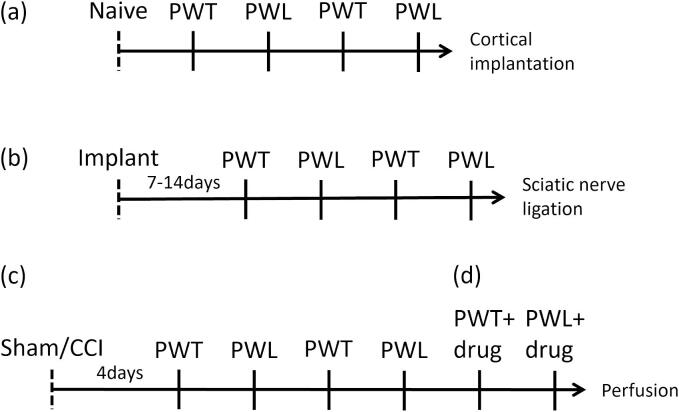
The illustration explains the time course of the experiments. Solid vertical ticks represent consecutive days. During experiments, mechanical (to measure paw withdrawal threshold, PWT) or thermal (to measure paw withdrawal latency, PWL) stimulus was applied to the hind paw under following conditions: (a) before any surgical manipulation, (b) after implantation of microinjection cannula (from 7–14 day after implantation), (c) after CCI or sham surgery (from 4 days after surgery), and (d) on microinjection. The two stimuli were applied on alternate days over four consecutive days except after microinjection. Further, each stimulus was applied alternately to the left and the right hind paw on the day of its application. The PWT was measured by applying 20–50 g mechanical stimulus six times to each paw in increments of 5 g. PWL was measured by applying 40 arbitrary units alternately to each paw at an inter-paw stimulus interval of at least 30 s. Each paw received five thermal stimuli. The selected drug or vehicle was microinjected twice on two separate days to examine the effects on PWT and PWL. The two microinjections were separated by a day during which the PWT was examined in absence of any treatment to demonstrate washout of drug effect on mechanical stimulation. The effect of microinjection on the PWT or the PWL was monitored from 15 min following the microinjection. The behavioural test was performed over a period of 30 min. The PWT and PWL, each, were grouped as follows: (i) Baseline: this is the average of animal responses in (a) and (b) above. Notably, the responses before (a) and after (b) implantation of cannula are not different from each other, (ii) Ligation: this is the average of animal responses recorded as in (c) above, and (iii) Microinjection: this is the responses recorded as in (d) above.

**Fig. 2:**
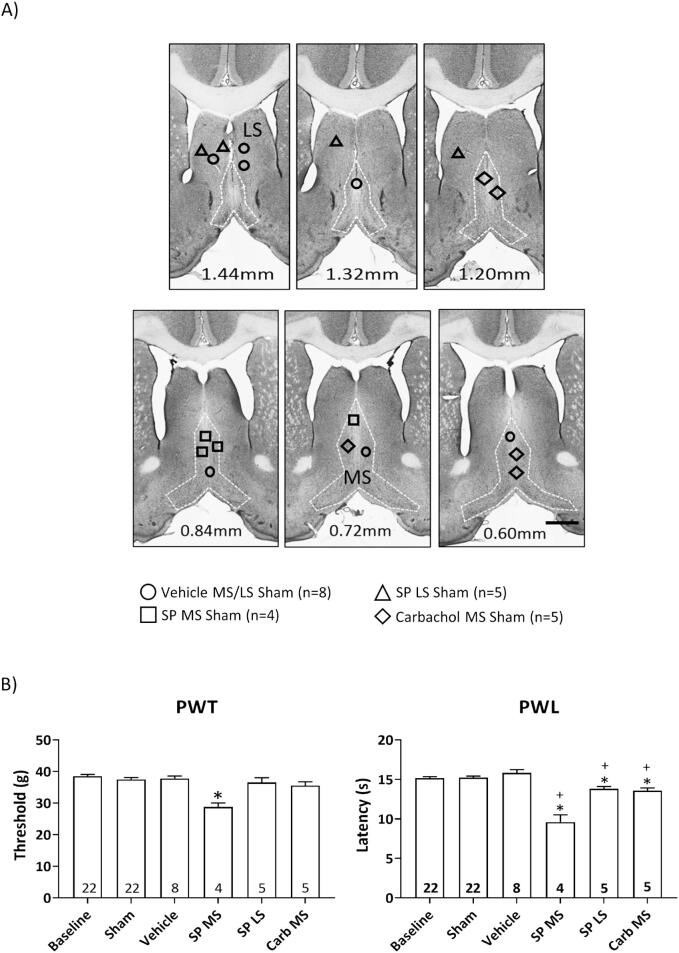
Intraseptal Substance P (SP) induces peripheral hypersensitivity-like effects. (A) Digital images through the septum depicting microinjection sites for SP (SP; 2 µg/µl, 0.5 µl), an agonist at neurokinin 1 receptor (NK1Rs), carbachol (0.156 µg/µl, 0.5 µl), a broad-spectrum cholinergic receptor agonist, or vehicle (saline, 0.5 µl) into the medial septum region (MS) or the lateral septal (LS) region. Numbers at bottom of each panel represents distance from Bregma. Scale bar represents 1 mm. The group nomenclature in the figure reflects the treatment administered and site of microinjection of the drug (or vehicle), and nature of ligation (sham surgery). Each drug or vehicle was microinjected in separate experiments. (B) Histograms representing hind paw withdrawal threshold (PWT; left) and paw withdrawal latency (PWL; right). The PWT and PWL were measured on alternate days as described in Fig. 1. The reflexes were measured in animals under the following experimental conditions: (a) before (‘Baseline’) and after sham ligation (‘Sham’), and (b) on microinjection of either vehicle (‘Vehicle MS/LS’), SP (‘SP MS’ or ‘SP LS’) or carbachol (‘Carbachol MS’) following sham surgery. 22 animals were used in the investigation. The baseline and post-Sham ligation responses were comparable across the experimental groups and, thus, combined (‘Baseline’, n = 22; ‘Sham’, n = 22). Similarly, the reflexive responses observed on vehicle microinjection into the MS or LS of sham animals were similar and, thus, combined (‘Vehicle’, n = 8). In sham animals, microinjection of SP into the MS induced bilateral hypersensitivity response to mechanical and thermal stimuli, whereas the microinjection of SP into LS or the microinjection of carbachol into MS evoked only thermal hypersensitivity that was, though, weaker as compared to that observed on microinjection of SP into MS. Data are presented as mean ± S.D. Significant difference (p < 0.05) (B) * vs. ‘Baseline’, + vs. ‘SP MS’; one-way ANOVA followed by Newman-Keuls post-hoc test.

**Fig. 3:**
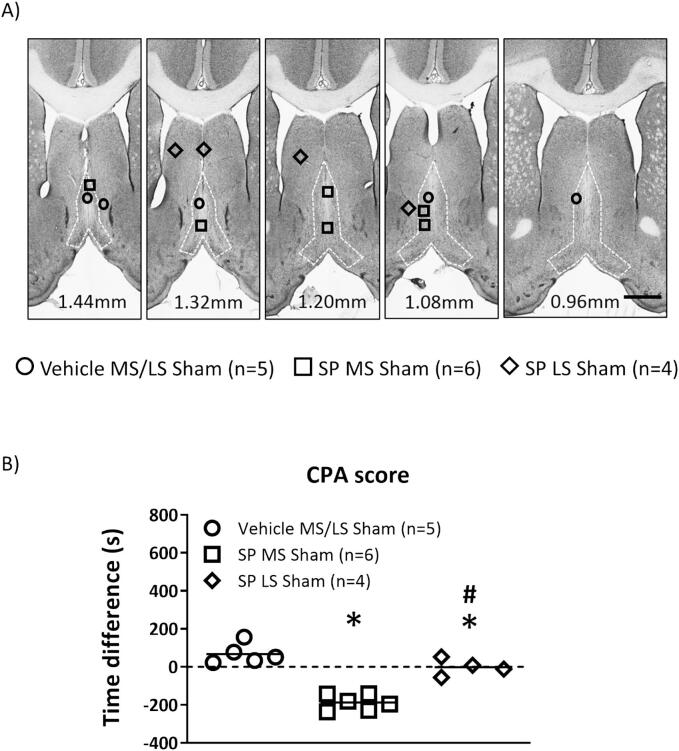
Microinjection of substance P (SP) into medial septal region (MS) evokes avoidance. (A) Digital images through the septum depicting microinjection sites for SP (SP; 2 µg/µl, 0.5 µl) or vehicle (0.5 µl) into the MS or the adjacent lateral septum (LS). Numbers at bottom of each panel represents distance from Bregma. Scale bar represents 1 mm. The group nomenclature reflects the treatment (Vehicle or SP, 0.5 µl) that was paired with exposure to the preferred compartment and the microinjection site (MS or LS) in sham ligated animals. (B) Scatter plot depicting animal conditioned place avoidance (CPA) on microinjection of drug. Animal avoidance was measured as the difference in time (‘Time Difference’) spent in the preferred compartment between the test (day 4) and the preconditioning (day 2) days of the CPA training. A negative score indicates avoidance behaviour. During conditioning, animals’ exposure to the non-preferred compartment was paired with microinjection of vehicle, while the exposure to the preferred compartment was paired with either microinjection of SP or the vehicle. Compared to Vehicle microinjection, SP in the MS elicited a robust place avoidance behaviour. A small but statistically significant avoidance was also observed on microinjection of SP into LS. Data presented as mean ± S.D. Significant difference (p < 0.05): * vs. ‘Vehicle MS/LS Sham’, # vs. ‘SP MS Sham’; one-way ANOVA followed by Newman-Keuls post-hoc test.

**Fig. 4:**
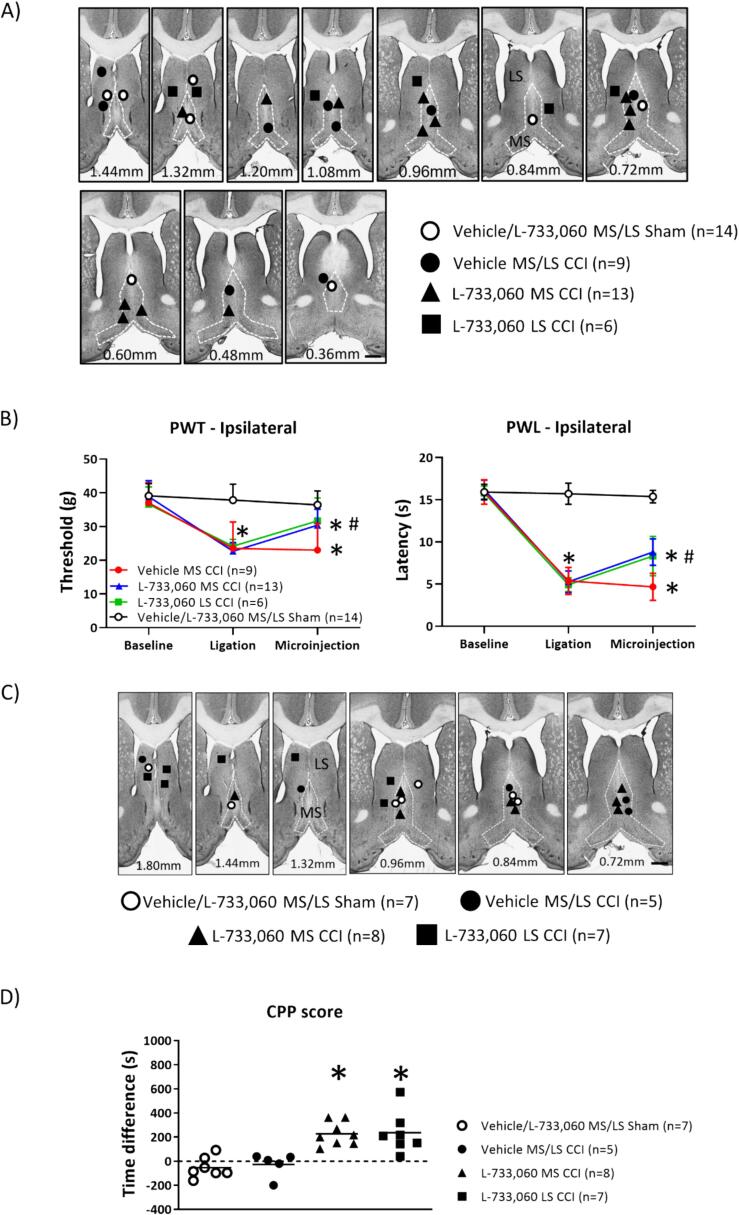
Intraseptal microinjection of L-733, 060, an antagonist at NK1R, attenuated peripheral hypersensitivity (PH) and evoked place preference in animals with CCI. (A) Digital images through the septum depicting microinjection sites for L-733,060 (0.0176 µg/µl, 0.5 µl) or vehicle (0.5 µl) into the medial septum region (MS) or the adjacent lateral septum (LS) in experiments testing PH in ‘Sham’ or ‘CCI’ animals. The number at bottom of each panel represents distance from Bregma. Scale bar represents 1 mm. (B) Line plot representing the effect of drug microinjection on paw withdrawal threshold (PWT; left panel) and paw withdrawal latency (PWL; right panel) to mechanical and thermal stimuli, respectively, when applied to the right hind paw ipsilateral to CCI. The group nomenclature and the building of the line plot is as described for Fig. 2. The PWT and PWL were measured as described in Fig. 1. Microinjection of L-733, 060 attenuated PH, increasing both the value of the PWT and PWL of the ipsilateral hind paw. (C) Digital images through the septum depicting microinjection sites for L-733,060 (0.0176 µg/µl, 0.5 µl) or vehicle (0.5 µl) into the MS or LS in experiments in (D). Scale bar represents 1 mm. The group nomenclature and the building of the plot is as described for Fig. 3. (D) Scatter plot depicting animal conditioned place preference (CPP) on microinjection of drug. The experimental protocol followed is as described for Fig. 3, except that during conditioning the first microinjection of vehicle was paired with the preferred chamber while the second microinjection of vehicle or L-733,060 was paired with the non-preferred chamber. The CPP score was computed as the difference in the times spent in the non-preferred chamber during CPP4 and CPP2. A positive score reflects a change in chamber preference. Note that microinjection of L-733,060 into either the MS or the LS induced CPP. Data presented as mean ± S.D. Significant difference (p < 0.05): (B) * vs. ‘Baseline’; # vs. corresponding value of the ‘Vehicle MS/LS CCI’ group; two-way RM ANOVA followed by Bonferroni post-hoc test; (D) * vs. ‘Vehicle/L-733,060 MS/LS Sham’ and ‘Vehicle MS/LS CCI’; one-way ANOVA with Newman-Keuls post-hoc test.

**Fig. 5:**
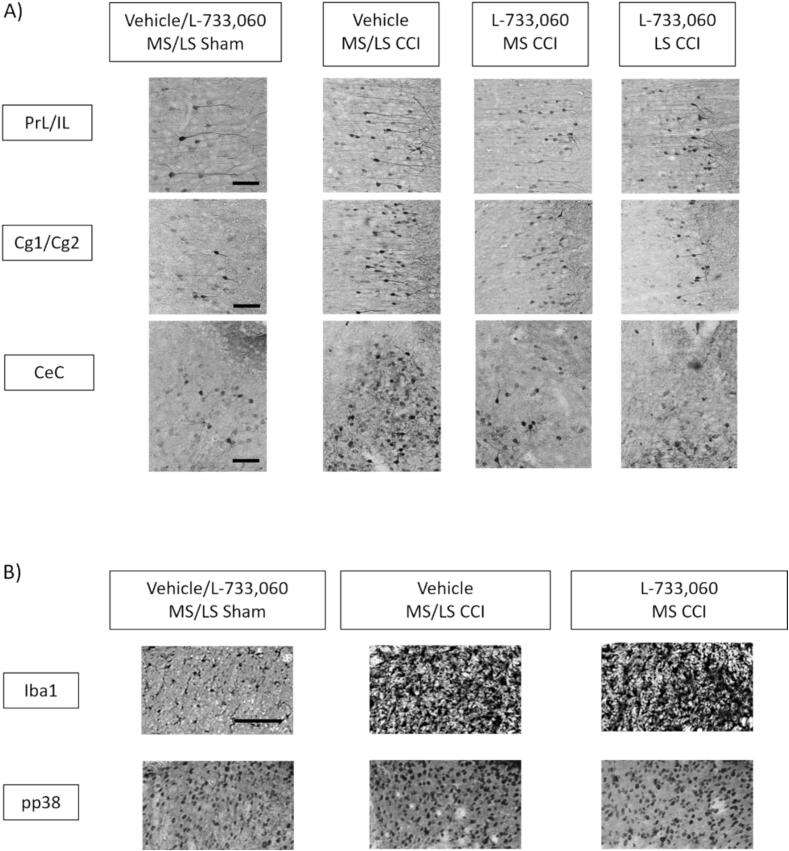
Pattern of expression of cellular markers on intraseptal microinjection of L-733, 060 in animals with CCI. The figure illustrates coronal sections through brain and spinal cord labelled for cellular markers using immunohistochemical techniques. The immunohistochemical labelling was performed on alternate sections taken through the regions of interest in the brain and the spinal cord of animals used in behavioural analysis of PWL in Fig. 4. Nomenclature of the groups is as in Fig. 4. (A) Digital images illustrating the expression of phosphorylated extracellular related kinase immunoreactivity (pERK-ir) in (i) the prelimbic/infralimbic (PrL/IL) and cingulate cortex areas 1 and 2 (Cg1/Cg2) and (ii) the capsular central amygdala (CeC). Note that pERK-ir neurons were intensely stained relative to the background. The contours of the neurons are outlined by the pERK-ir since the kinase is expressed in cytoplasm. (B) Digital images illustrating the expression of phosphorylated p38 (pp38)-ir and ionized calcium binding adaptor molecule 1 (Iba1)-ir in the L4 region of the lumbar spinal cord. Note the intense Iba1-ir and the increase in punctate pp38-ir in the spinal cord of animals with CCI. Scale bars: PrL/IL and Cg1/2, 50 µm; CeC, 500 µm; Spinal cord, 100 µm.

**Fig. 6:**
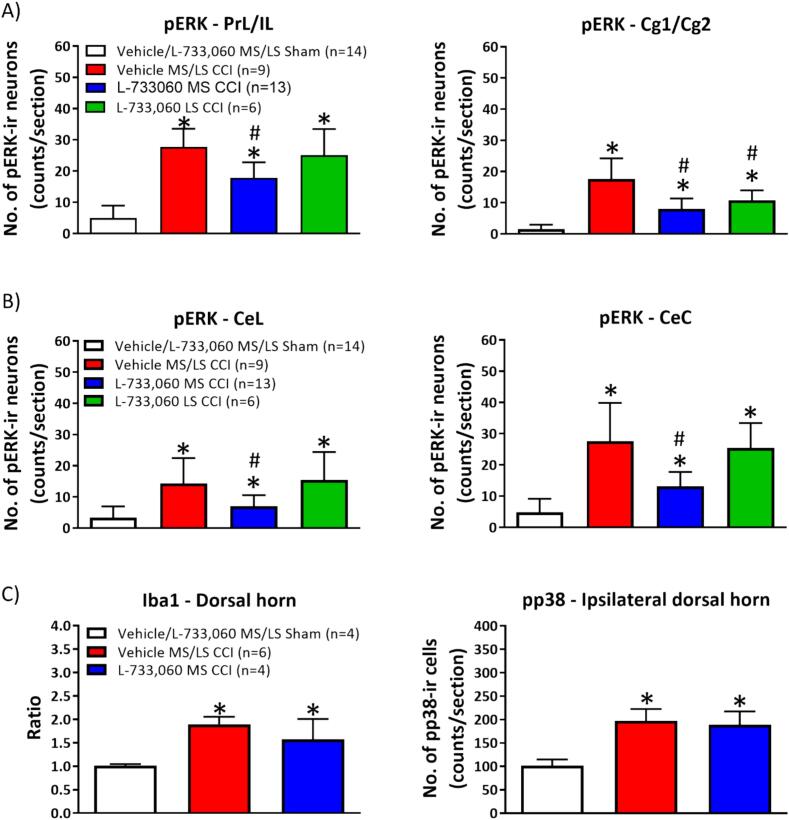
Intraseptal L-733,060 selectively modulates cellular response in the forebrain of animals with CCI. (A, B) The number of positively labelled pERK-ir cells were averaged bilaterally in each section and again across the total number of sections analysed from each animal. The boundaries of the different regions were demarcated based on the rodent atlas. (A) Histograms of the average number of pERK-ir neurons per section of prelimbic/infralimbic (PrL/IL; left panel) and cingulate areas 1 and 2 (Cg1/Cg2; right panel) of the medial prefrontal cortex (mPFC) from animals as in Fig. 4. The regions were labelled immunohistochemically as described in Fig. 5. (B) Histograms of the average numbers of pERK-ir neurons per section of lateral (CeL; left panel) and capsular (CeC; right panel) regions of the central amygdala in different groups of animals. (C) Histograms of the relative density of Iba1-ir (left panel) and the number of cells expressing pp38-ir (right panel) in the dorsal horn (lamina I-IV) of the lumbar spinal cord ipsilateral to CCI or sham ligation. Iba1-ir, being amorphous, was analysed as a ratio of the relative optical density (ROD) of the ipsilateral to the contralateral dorsal horn of each section of the lumbar spinal cord. The ratios were averaged across the number of sections analysed from each animal. The number of pp38-ir cells were averaged across the number of sections analysed from each animal. While CCI alone increased the levels of the different cellular markers in the forebrain and the spinal cord, intraseptal microinjection of L-733, 060 selectively decreased expression in the forebrain. Microinjection into the MS region had a more widespread effect than the microinjections into the LS. Data are presented as mean ± S.D. Significant difference (p < 0.05): * vs. ‘Vehicle/L-733, 060 MS/LS Sham’, # vs. ‘Vehicle MS/LS CCI’; one-way ANOVA followed by Newman-Keuls post-hoc test.

**Fig. 7:**
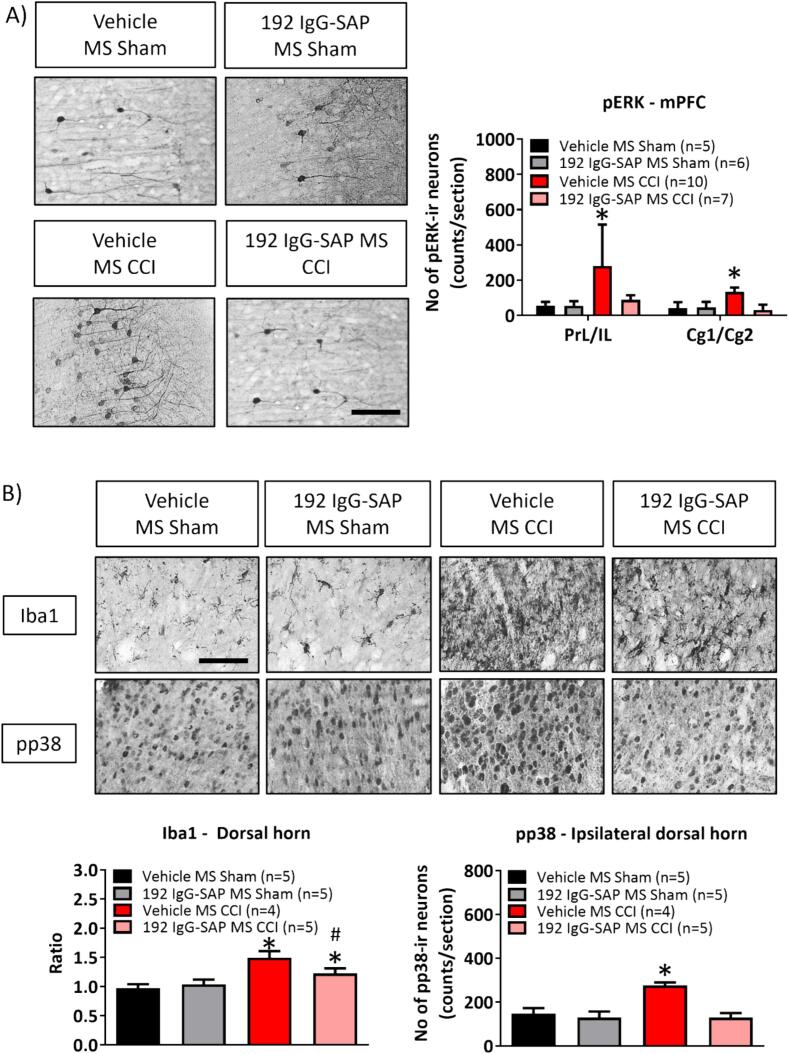
Destruction of septal cholinergic neurons attenuate peripheral hypersensitivity (PH) in the CCI model. The animals were pre-treated with either the neurotoxin 192 IgG-Saporin (192 IgG-SAP; 0.42 µg/µl, total of 2 µl) or equivalent volume of vehicle (saline). The group nomenclature in the figure reflects the treatment administered and site of microinjection of the drug (or vehicle), and nature of ligation (sham or CCI surgery). The animals were subject to behavioural evaluation at different stages of the experiment, i.e., ‘Baseline’, ‘Lesion’, and ‘Ligation’ at end of which the brain and spinal cord of animals were prepared for immunohistochemistry as illustrated in (A) and (B). (A) The panels on the left are digital images of pERK–ir neurons in sections taken through the PrL/IL region of the mPFC of the different experimental groups, while the histogram on right illustrates the number of pERK-ir cells in the mPFC from various groups of animals. (B) Top panels are digital images of Iba1-ir and pp38-ir cells in sections through spinal dorsal horn, while the histograms below illustrate the changes in the levels of these markers in various groups of animals. Note that cholinergic lesion with 192 IgG-SAP prevented the increase in pERK-ir on CCI and either attenuated or prevented the increase in spinal markers on CCI as compared to vehicle pre-treated CCI animals. Data presented as mean ± S.D. Significant difference (p < 0.05): (A) and (B) * vs. ‘Vehicle MS Sham’, # vs. ‘Vehicle MS CCI’; one-way ANOVA followed by Newman-Keuls post-hoc test. Scale bar represents 50 µm.

**Fig. 8:**
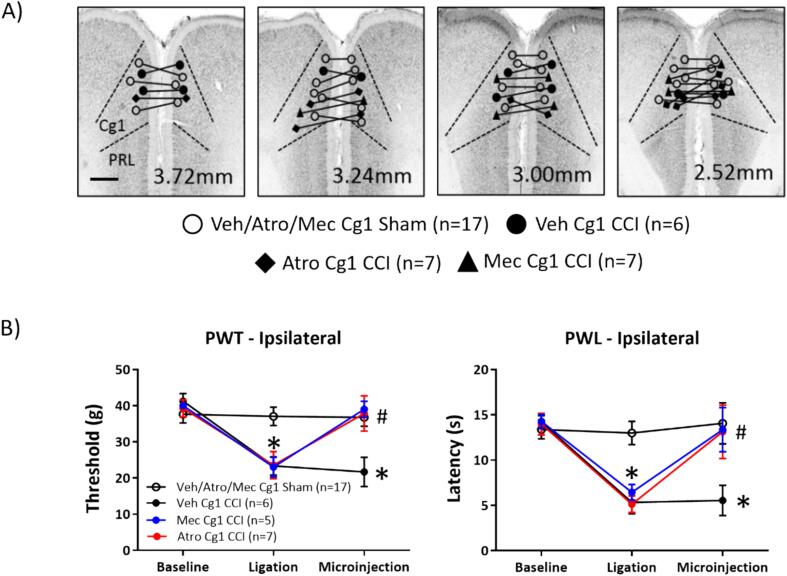
Microinjection of cholinergic receptor antagonists into cingulate cortex (Cg) attenuated peripheral hypersensitivity (PH) in the CCI model. (A) Digital images depicting the location of the bilateral microinjection sites in the Cg1 region. The lines connecting the symbols on the digital images indicate the bilateral microinjection sites matched by experiments. Number at bottom of each panel represents distance from Bregma. Scale bar represents 1 mm. The cholinergic muscarinic receptor antagonist, atropine (‘Atro’; 0.007 µg/µl, 0.5 µl per site), the cholinergic nicotinic receptor antagonist, mecamylamine (‘Mec’; 2 µg/µl, 0.5 µl per site), or the vehicle (Veh; 0.5 µl per site) were microinjected bilaterally in separate experiments. (B) Line plot representing the effect of drug microinjection on paw withdrawal threshold (PWT; left panel) and paw withdrawal latency (PWL; right panel) to mechanical and thermal stimuli, respectively, applied to the right hind paw ipsilateral to CCI or sham ligation. The animals were subject to behavioural evaluation at different stages of the experiment, i.e., ‘Baseline’, ‘Ligation’ and ‘Microinjection’. The line plot is built as described for Fig. 2. The PWT and PWL were measured on alternate days as described in Fig. 1. Note that cholinergic antagonists decrease PH in the CCI model. Data presented as mean ± S.D. Significant difference (p < 0.05): * vs. ‘Baseline’; # vs. corresponding value of the ‘Vehicle Cg1 CCI’ group; two-way RM ANOVA followed by Bonferroni post-hoc test.

**Fig. 9:**
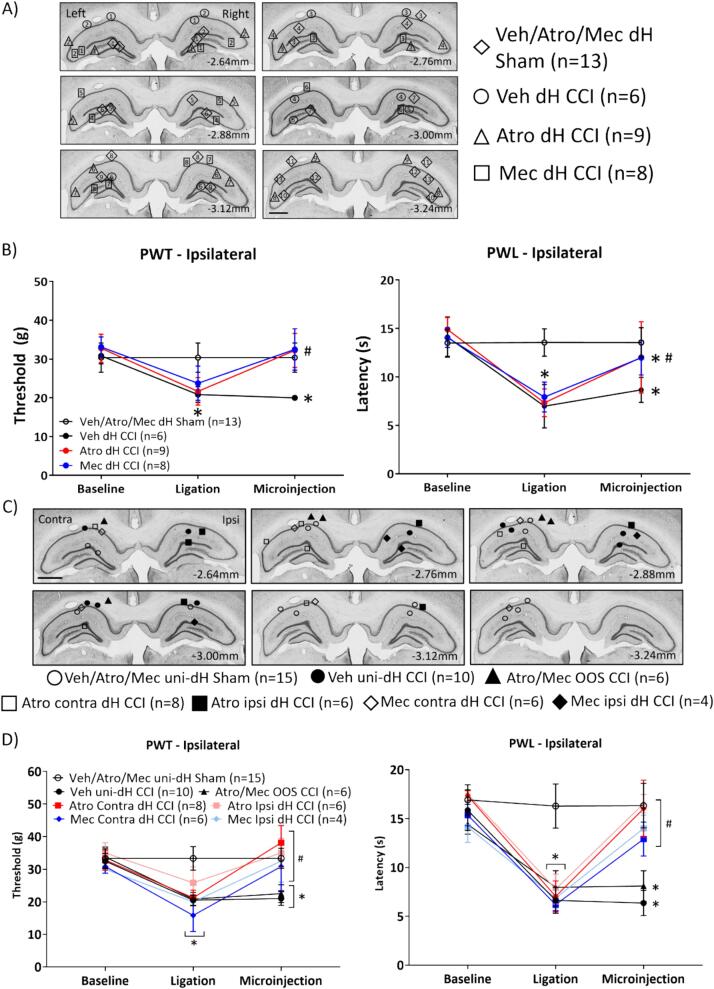
Bilateral or unilateral microinjection of cholinergic receptor antagonists into the dorsal hippocampus (dH) attenuated peripheral hypersensitivity (PH) in the CCI model. Experimental groups are identified by the treatment, microinjection site, and nature of ligation. (A) Digital images depicting the location of the bilateral microinjection sites in the dH. The pair of microinjection sites in a given experiment of the experimental group are identified by the matching numbers within each symbol representing the experimental group. The microinjection sites were distributed in all hippocampal fields. The drugs microinjected are as in Fig. 8. Effects of different microinjections in sham ligated animals were similar and as such combined to from the ‘Veh/Atro/Mec dH Sham’ group. (B) Line plot representing the effect of drug microinjection on paw withdrawal threshold (PWT; left panel) and paw withdrawal latency (PWL; right panel) to mechanical and thermal stimuli, respectively, when applied to the right hind paw ipsilateral to CCI or sham ligation. The animals were subject to behavioural evaluation at different stages of the experiment, i.e., ‘Baseline’, ‘Ligation’ and ‘Microinjection’. The line plot is built as described for Fig. 2. The PWT and PWL were measured on alternate days as described in Fig. 1. Compared to vehicle (Veh) treated animals, atropine (Atro) or mecamylamine (Mec) administered bilaterally in dH reversed CCI-induced PH in the ipsilateral hind paw. (C) Digital images depicting location of unilateral microinjection sites into the contralateral (Contra, left dH) or ipsilateral (Ipsi, right dH). In some CCI-ligated animals, microinjections were made in region outside of the dH (‘Atro/Mec OOS CCI’ group). The responses to microinjection of vehicle, atropine, or mecamylamine in sham animals were similar and thus combined to form the ‘Veh/Atr/Mec uni-dh Sham’ group. Similarly, animals receiving vehicle in the ipsilateral or contralateral dH were combined to form the ‘Veh uni-dH CCI’ group. In the remaining CCI groups, the site of microinjection is identified as ipsilateral (ipsi) dH or contralateral (contra) dH. Scale bar represents 1 mm. (D) Line plot illustrating the effect of unilateral microinjections on PH. Microinjection of cholinergic antagonists into either the contralateral or ipsilateral dH increased the PWT or PWL values towards sham ligated group. Data presented as mean ± S.D. Significant difference (p < 0.05): * vs. ‘Baseline’ values; # vs. corresponding value of the sham ligated group; two-way RM ANOVA followed by Bonferroni post-hoc test.

**Fig. 10:**
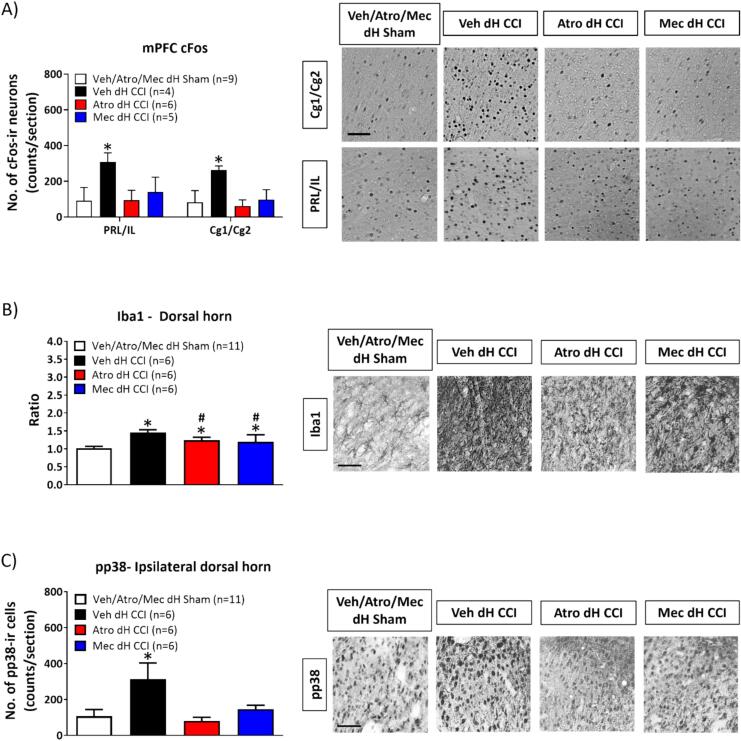
Bilateral microinjection of cholinergic receptor antagonists into the dorsal hippocampus (dH) modulated CCI-induced cellular changes. Experimental groups are defined as in Fig. 9. The brain and spinal cord of animals used in the behavioural tests above (Fig. 9) were prepared for immunohistochemistry after the last test of PWL and probed as follow: (A) c-Fos-immunoreactivity (ir) in the medial prefrontal cortex (mPFC), (B) Iba1-ir and (C) pp38-ir in the lumbar spinal cord. Histograms (left panel) depict the average number of immunoreactive neurons per tissue section of different CNS regions. The corresponding representative micrographs from different experimental group are shown on right of the histograms. 10–15 coronal sections through the mPFC, and lumbar spinal cord were analysed. The number of positively immunolabelled structures were averaged across the number of sections analysed and across the number of animals in each experimental group. Data presented as mean ± S.D. Significant difference (p < 0.05): * vs. ‘Veh/Atro/Mec dH Sham’, # vs. ‘Veh dH CCI’; one-way ANOVA followed by Newman-Keuls post-hoc test. Scale bar represents 50 µm.

**Fig. 11:**
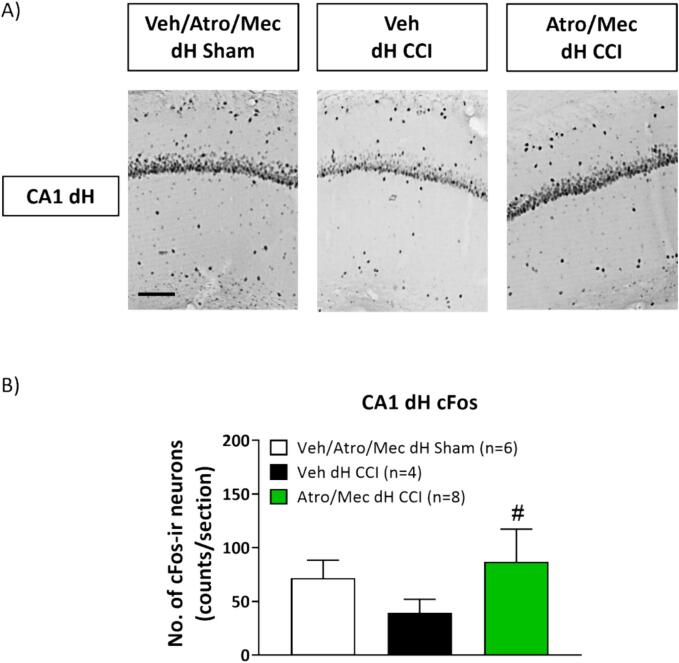
Bilateral microinjection of cholinergic receptor antagonists into the dorsal hippocampus (dH) modulated CCI induced cellular changes. c-Fos-immunoreactivity (ir) was computed from experimental groups as described in Fig. 10. 10–15 coronal sections through the dH were analysed. The group of animals with CCI-ligation that received atropine or mecamylamine were combined. (A) Representative micrographs at high magnification of the CA1 field in the dH. (B) Histogram depicting the average number of immunoreactive neurons per tissue section. Data presented as mean ± S.D. Significant difference (p < 0.05): # vs. ‘Veh dh CCI’; one-way ANOVA followed by Newman-Keuls post-hoc test. Scale bar represents 100 µm.

*Pharmacological investigations in behaving rat (*Fig. 1, Fig. 2, Fig. 3, Fig. 4, Fig. 5, Fig. 6, Fig. 7, Fig. 8, Fig. 9, Fig. 10, Fig. 11*):* Investigations were undertaken to determine the effects of intracerebral administration of drugs on CCI-induced PH. Such experiments were performed between days 4–17 after sham surgery or CCI. Notably, the PH reaches a peak by day 4 post-ligation and is stable thereafter (Ariffin et al., 2018). The behavioural test was performed over a period of 30 min. It is notable here that electrophysiological evidence published earlier by the laboratory indicates that the effects of drugs used in the current study are sustained on their microinjection. The period of this drug effect overlaps with the period of behavioural observations in the present study (Ariffin et al., 2010, Ariffin et al., 2017, Ibrahim et al., 2021, Jiang and Khanna, 2006, Ng et al., 2021). The time-course of the experiments and the protocol followed is explained in Fig. 1.

In a deviation from procedure described in Fig. 1, PWT and PWL were also recorded in an experiment paired with i.p. administration of drug or vehicle preceding the intracerebral microinjection of drug (also see below). While, in context of Fig. 7, neurons were lesioned with intracerebral microinjection of an immunotoxin, instead of receiving an implant. The PWT or the PWL measured after microinjection of the immunotoxin is labelled as ‘Lesion’ (also see below).

The pharmacological treatment tried in each experiment is identified in the nomenclature used to describe the experimental group. Typically, the groups are named as follows: drug treatment that included i.p. administration and/or microinjection, site of microinjection, Sham or CCI surgery. The following pharmacological investigations were undertaken in awake animal:

1. Investigation into the effect of intraseptal SP on animal reflexive responses to peripheral stimuli (Fig. 2): The protocol followed is as described in Fig. 1. The effect of SP (2 µg/µl, 0.5 µl) or equivalent volume of vehicle was also compared with intraseptal microinjection of carbachol (0.156 µg/µl, 0.5 µl). The drugs were microinjected in separate experiments. The effect of microinjection was examined on PWT and PWL on days 12 and 14, respectively, post sham surgery. At the dose used, intraseptal carbachol induces a robust septo-hippocampal activation marked by a robust suppression of CA1 PS and theta activation, the suppression with carbachol being like that seen with 2 µg/µl of intraseptal SP (Ibrahim et al., 2021, Ng et al., 2021, Zheng and Khanna, 2001)

In total, 22 animals comprised this investigation. The animals were randomly distributed to the following groups: Vehicle MS/LS Sham (n = 8), SP MS Sham (n = 4), SP LS Sham (n = 5), and Carbachol MS Sham (n = 5). The baseline response of all were similar and grouped together (‘Baseline’ with open bar in Fig. 2B, n = 22). Likewise, the animal responses after sham surgery were similar and grouped together (‘Sham’ in Fig. 2B, n = 22). The preceding ‘Baseline’ and ‘Sham’ responses were compared with animal responses on microinjection of vehicle, SP or carbachol.

As a measure of the strength of change in PWT and PWL with drug injections, the drug-induced responses were compared with changes in PWT and PWL on vehicle microinjection following CCI. CCI ligation was performed in a separate group of animals (n = 9). The behavioural observations were made on days 7 and 9 (for PWT) and 8 and 10 (for PWL) after CCI ligation. While the observation corresponding to ‘Vehicle’ were made on days 12 and 14 after ligation. The ‘Baseline’ response of this group of ligated animals was also compared with that of other groups in the study.

2. Investigation into the effect of atropine i.p. on intraseptal SP-induced changes in PWT and PWL: In a separate group of animals, atropine (5 mg/kg, i.p.) was administered prior to intraseptal microinjection of SP. As above, the reflexive responses were monitored at baseline, on sham ligation, 30 min following administration of atropine or vehicle, and on SP microinjection. SP was microinjected into the MS region 35–40 min after i.p. Administration.

3. Effect of intraseptal SP in CPA (Fig. 3): The place preference experiment was performed on days 14–17 after sham ligation. The groups of animals were as follows: Vehicle MS/LS sham (n = 5), SP MS sham (n = 6), SP LS sham (n = 4). As the first microinjection is always vehicle paired with the non-preferred chamber, the group nomenclature indicates the treatment that was paired with exposure to the preferred compartment

4. Effect of intraseptal L-733,060 on CCI-induced PH (Fig. 4A, B, 5 and 6): The protocol involved in investigating the effect of the NK1R antagonist, L-733,060, on PH is as described in Fig. 1. The groups included in this investigation are as follows: Vehicle MS/LS sham (n = 14), vehicle MS/LS CCI (n = 9), L-733,060 MS CCI (n = 13), L-733,060 LS CCI (n = 6). The animals were sacrificed immediately after the test of PWL, and the brain and spinal cord fixed for later histology (Fig. 5, Fig. 6; see below)

5. Effect of intraseptal L-733,060 in CPP (Fig. 4C, D): The CPP protocol was carried out from day 14 to day 17 after ligation. The following groups were included in the investigation: Vehicle/L-733,060 MS/LS sham (n = 7), vehicle MS/LS CCI (n = 5), L-733,060 MS CCI (n = 8), L-733,060 LS CCI (n = 7). Prior to CPP, the animals were tested for PH to verify that the ligation was effective in altering sensory responses

6. Effect of immunotoxin pre-treatment on CCI-induced PH (Fig. 7): The immunotoxin 192 IgG-SAP (0.42 µg/µl, total volume of 1 µl) was microinjected into the MS region to lesion the cholinergic neurons in the region. The baseline behavioural response (‘Baseline’) was recorded in naïve animals prior to microinjection. The effects of microinjection of the immunotoxin or vehicle *per s*e (‘Lesion’) were recorded on days 14–17 days after microinjection and that following CCI or sham surgery in microinjected animals (‘Ligation’) were recorded on days 4–7 after CCI/sham surgery. The brain and spinal cord were taken out immediately after the test of PWL on day 7 after CCI/sham surgery for immunohistochemical analysis (Fig. 7A, B). The following groups comprised this study: Vehicle MS sham (n = 8), vehicle MS CCI (n = 13), 192 IgG-SAP MS sham (n = 11) and 192 IgG-SAP MS CCI (n = 12)

7. Effect of microinjection of atropine or mecamylamine into cingulate cortex on CCI-induced PH (Fig. 8): The cholinergic antagonists, atropine (Atr; 0.007 µg/µl, 0.5 µl) or mecamylamine (Mec; 2 µg/ml, 0.5 µl) or vehicle (Veh, 0.5 µl) was administered in separate experiments. The drug or vehicle were administered bilaterally into cingulate area 1 (Cg1) of the mPFC (Paxinos and Watson, 2013). The test for PH was performed from 15 min after drug microinjection. The experimental groups in this investigation are Veh/Atr/Mec Cg1 sham (n = 17), Veh Cg1 CCI (n = 6), Atro Cg1 CCI (n = 7), and Mec Cg1 CCI (n = 5)

8. Effect of microinjection of atropine or mecamylamine into dH on PH (Fig. 9, Fig. 10, Fig. 11): Vehicle (Veh; 0.5 µl) or the cholinergic antagonist atropine (Atr; 0.007 µg/µl, 0.5 µl) or mecamylamine (Mec; 2 µg/l, 0.5 µl) was microinjected into the dH bilaterally (Fig. 9A, B) or unilaterally in the ipsilateral (ipsi) or contralateral (contra) dH (Fig. 9C, D). The ipsilateral or contralateral dH corresponds to the right and the left dH respectively, the ipsilateral dH being on the same side as the Sham/CCI surgery. Behavioural tests were conducted from 15 mins after microinjection. The brain and spinal cord were taken out immediately after the last test of PWL in the experiments involving bilateral microinjections for immunohistochemical analysis (Fig. 10, Fig. 11). The experimental groups receiving bilateral microinjections in the dH are as follow: Veh/Atro/Mec dH sham (n = 13), Veh dH CCI (n = 6), Atro dH CCI (n = 9), and Mec dH CCI (n = 8). The experimental groups that received unilateral microinjections in the dH are as follow: Veh/Atro/Mec ipsi/contra dH sham (n = 15), Veh ipsi/contra dH sham (n = 10), Atro contra dH CCI (n = 8), Atro ipsi dH CCI (n = 6), Mec contra dH CCI (n = 6), and Mec ipsi dH CCI (n = 4). Additionally, in some animals, unilateral microinjection of Atro (n = 3) or Mec (n = 3) were localised to the corpus callosum overlying the dH i.e., out of site (OOS). These animals were consolidated to form the Atro/Mec OOS CCI group (n = 6) as a site control.

### 2.7 Histology

Immediately after the last behavioral test, animals were deeply anaesthetized with urethane (1.5 g/kg, i.p.; Sigma, USA) and transcardially perfused with 4 % paraformaldehyde in 0.1 M phosphate buffer (Sigma, USA). 60 µm and 40 µm coronal sections of the brain and spinal cord, respectively, were made on the vibratome (Leica VT1200, Leica Microsystems GmbH, Germany).

The protocol for immunohistochemical staining was as described previously (Ang et al., 2015, Ariffin et al., 2018). Alternate serial sections through the dH and mPFC were collected and incubated for 72 hrs with rabbit anti-cFos (1:2500, #2250, Cell Signaling USA). In other experiments, alternate sections through amygdala and/or mPFC were incubated with rabbit anti-pERK (1:1500, #9101, Cell Signaling USA) antibody (Fig. 5, Fig. 6, Fig. 7). Alternate serial sections through L4 lumbar spinal cord were incubated with rabbit anti-Iba1 antibody (1:2000, #019–19741, Wako, Japan) and rabbit anti-pp38 antibody (1:500, #4511, Cell Signaling, USA), respectively (Fig. 5, Fig. 6, Fig. 7). In animals that underwent septal cholinergic lesions, alternate serial sections through the MS were collected and incubated with goat anti-ChAT (1:400, AB144P, Millipore USA) or mouse anti-PV (1:2000, P3088, Sigma, USA) antibody, ChAT and PV being markers of cholinergic and GABAergic septal neurons, respectively. Subsequently, the sections were incubated overnight with the corresponding biotinylated secondary goat anti-rabbit (1:1000, B7389, Sigma, USA), goat anti-mouse (1:400, sc516142, Santa Cruz, USA), or mouse anti-goat (1:400, sc2489, Santa Cruz, USA) antibodies. The horseradish peroxidise-avidin–biotin complex (ABC, Vector Laboratories Inc., USA) was used to detect the antigen using diaminobenzidine as the chromogen. Labelled sections were mounted on gelatin coated slides and cover slipped. Omission of the primary antibody led to a complete loss of labelling, suggesting that the secondary antibody did not cross-react with tissue components (secondary antibody control) and that the tissue did not contain functional enzymes to convert the substrates (labelling control).

Sections through the MS, mPFC, or dH containing the cannula track(s) were mounted on gelatin coated slides, air dried, and stained with Cresyl violet (0.5 % w/v; Sigma, USA) to identify the microinjection sites.

### 2.8 Data analysis

*Analysis of electrophysiological recordings*: In experiments investigating the effect of intraseptal microinjection of SP on PS, the PS amplitude (mV) was calculated as the average amplitude between the negative peak from the 2 positive peaks around it as described previously (Ng et al., 2021). PS amplitude was averaged over six sweeps in 1 min blocks. The magnitude of PS amplitude reflects the size of the neuronal population discharging synchronously in response to CA3 stimulation.

Furthermore, our published evidence suggests that the effect of intraseptal SP reaches a maximal in the first 5 min following microinjection. As a result, we compared the average PS amplitudes in that period for the different groups, the average amplitude being expressed as a percentage of the ‘control’ PS amplitude, the ‘control’ PS amplitude being the average amplitude in the two-minute preceding microinjection.

*Quantification of nociceptive behaviors:* The animal withdrawal of hind paw was signalled by flinching or lifting of the tested paw within 6 s and 20 s of the application of the mechanical and thermal stimuli, respectively. The time of paw lift was automatically recorded by the anesthesiometer/plantar apparatus. The PWT (g) for each paw was the mechanical stimulation intensity that elicited at least 4 paw withdrawals out of 6 applications. The PWL (s) was measured as the average time taken to withdraw paw to 5 applications of peripheral thermal stimulation. The PWT and PWL for each phase of pH experiment were averaged for a given animal and then averaged across the group.

*Analysis of place conditioning*: The CPA score was the difference in the time spent in the preferred chamber during the test session CPA4 and the pre–conditioning session CPA2. A negative score reflects avoidance behaviour. The CPP score was the difference in the time spent in the non-preferred chamber during CPP4 and CPP2. In this instance, a positive score reflects a change in chamber preference.

*Quantification of immunolabeling*: Immunolabeled sections were digitized at a resolution of 1280 x 960 pixels under 4x magnification (Nikon Eclipse Ci microscope with attached DS-F12 camera; Nikon Corporation, Japan) using the NIS Elements software. Immunoreactivity (ir) was quantified with the MCID software (Interfocus Imaging Ltd, Niagara Inc, USA). As previously (Ang et al., 2015), the following parameters were adopted for analysis:

i) the intensity of immunolabeled neurons was at least 180 % more intense than the background intensity through the region,

ii) the area of the label was ≥ 5 pixels for cFos- and pp38- ir, or ≥ 10 pixels for pERK-, PV- and ChAT-ir. Notably, cFos- and pp38-ir are nuclear labels and appear as smaller spherical structures in contrast to pERK-, ChAT- and PV-ir which stains the cytoplasmic space. Thus, the minimal pixel size to identify positive labelling is smaller for cFos and pp38 as compared to pERK, ChAT and PV, and.

iii) form factor of 0.8 (form factor is a measure of degree of roundness, 1 indicates a perfectly round target). Here it is notable that we performed a pilot analysis wherein ten random sections through the mPFC were selected and analysed manually or digitally using two different form factors – 0.5 and 0.8. The average number of pERK immunoreactive neurons per section for manual count vs. digital count with form factor of 0.5 vs. digital count with form factor of 0.8 was as follows (mean ± SD): 152.1 ± 27.14 (range: 120–192) vs. 138.6 ± 25.81 (range 107–182) vs. 144.9 + 25.78 (range: 120–176). The counts were not significantly different from each other (Groups, F_2,27_ = 0.66, p > 0.5; one-way ANOVA). The manual count tended to be higher than the digital counts since some of lightly stained cells were not counted by digital means, presumably because these cells did not match the intensity threshold set for digital count. Crucially, however, the cells counted digitally were acceptable on visual screening as stained for the marker. Furthermore, the manual count correlated tightly with digital count (Pearson correlation coefficient, r (18) = 0.93, p < 0.0002 for manual vs. digital count with form factor of 0.5, and r (18) = 0.95, p < 0.0001 for manual vs. digital count with form factor of 0.8), suggesting that digital count varied tightly with the manual count from section to section. Following from this we analysed pERK-ir with form factor of 0.8, which is also consistent with our previous approach (Ariffin et al., 2018).

Iba1-ir was quantified by measuring the relative optical density (ROD) since a dense immunolabeling was detected which made it difficult to distinguish individual cells. The ROD was determined by the analytical software by computing the average grey level intensity per pixel. The number of pixels was determined by the size of the demarcated area i.e., dorsal horn spinal cord. A larger ROD value indicates a higher intensity stain.

The number of cFos-ir or pERK-ir neurons in the subregions of the mPFC (AP 4.20 to −0.48 mm from Bregma; 15–20 sections), amygdala (AP −1.92 to −3.24 mm from Bregma; 9–11 sections) or dH subfield CA1 (AP −2.16 to −5.64 mm from Bregma; 10–15 sections) were quantified and averaged bilaterally and subsequently across the total number of sections analysed. The number of ChAT- and PV-ir neurons in the septal region (AP 1.80 to 0.0 mm from Bregma; 8–15 sections) were quantified in a similar fashion. Analysis of pp38-ir and Iba1-ir in the lamina I to IV of the spinal cord dorsal horn region (12–20 sections) was based on demarcation of the grey matter according to Molander et al. (Molander et al., 1984). The number of pp38-ir cells were quantified in the ipsilateral dorsal horn, while the relative density of Iba1-ir was computed by taking the ratio of the ROD of the ipsilateral to contralateral dorsal horn. The cell counts or the relative density was then averaged across the sections analysed. The sections were taken from different animals. The number of animals analysed (or the ‘n’ number) are identified with the presentation of results in Fig. 6, Fig. 7, Fig. 10, Fig. 11. In addition, the ‘n’ value corresponding to cellular data presented in the text is presented alongside that data (see Last paragraph of the sub-section ‘Effect of bilateral microinjection of cholinergic receptor antagonists on cellular changes in the CCI-model’ under section ‘Decreasing septo-hippocampal cholinergic function strongly attenuates CCI-induced nociception’ of the ‘Results).

### 2.9 Statistical analysis

Results are expressed as mean ± S.D and were analyzed using Prism (GraphPad Software Inc.). For comparison across multiple experimental groups and time-points, data were analyzed using repeated measure two-way ANOVA followed by Bonferroni post-hoc test. Comparison between multiple groups was performed with one-way or one-way repeated measures (RM) ANOVA followed by Neuman-Keuls post-hoc test. In the event of a significant Bartlett’s test of assumed normal distribution, the data sets were transformed, and the resultant values subjected to ANOVA analysis. Alternatively, the non-parametric Kruskal-Wallis test with post-hoc Dunn’s test was carried out. Two-group analysis was carried out using two-tail paired or unpaired *t*-test, while correlation was determined using Pearson correlation coefficient. Statistical significance was accepted at p ≤ 0.05.

## Results

***Intraseptal microinjection of SP promotes aversive behaviour mediated by cholinergic muscarinic receptor mechanisms***.

General: These experiments were performed in awake animals to examine the effect of intraseptal SP, a ligand at NK1R, on nociceptive behaviours vs. the nociceptive effect of intraseptal carbachol, a broad-spectrum cholinergic receptor agonist. Notably, while the NK1R are expressed exclusively in cholinergic neurons, the cholinergic receptors, including M2 receptors, are expressed in both cholinergic and non-cholinergic neuropil in the MS region, although the expression is stronger and more extensive in non-cholinergic neurons vis-à-vis the cholinergic neurons (Levey et al., 1995, Van der Zee and Luiten, 1994). Indeed, the cholinergic agonists and endogenous acetylcholine directly excite local GABAergic and glutamatergic neurons, also implicated in nociception, but not cholinergic neurons (Alreja et al., 2000, Alreja et al., 2000, Ang et al., 2015, Fan et al., 2023, Ibrahim et al., 2021, Wu et al., 2003, Wu et al., 2003, Wu et al., 2000; Zheng and Khanna, 2001). However, carbachol might indirectly excite septal cholinergic neurons through intraseptal glutamatergic mechanisms to modulate excitability of hippocampal field CA1 pyramidal cell (Ibrahim et al., 2021, Zheng and Khanna, 2001). On the other hand, SP directly excites septal cholinergic neurons to modulate excitability of CA1 pyramidal cell (Morozova et al., 2008, Ng et al., 2021). Interestingly, carbachol, unlike SP, also evokes robust hippocampal theta rhythm, in part due to excitation of septal GABAergic and glutamatergic neurons, theta being extracellular oscillatory activity reflecting a synchronized processing of information in the septo-hippocampus neural network (Bender et al., 2015, Fan et al., 2023, Fuhrmann et al., 2015, Ng et al., 2021). Thus, this experiment with intraseptal SP allowed us to examine the selectivity of NK1R, localized exclusively on septal cholinergic neurons, in evoking nociception vs. the effect of a more broad-based excitation of MS with carbachol.

Experimenters were blind to drug administered to animals.

Intraseptal SP induces a ‘V’ shaped suppression: In context of SP, comparison of the average amplitude of PS in CA1 showed that microinjection of the three doses of SP induced a suppression as compared to the control (p < 0.0009; Kruskal-Wallis test followed by Dunn’s post-hoc test). In this context, the values of Control – Saline vs. SP 1 µg/µl vs. SP 2 µg/µl vs. SP 7 µg/µl are as follows: 96.51 ± 1.80 % (n = 5) vs. 60.70 ± 18.48 % (n = 7) vs. 38.84 ± 12.88 % (n = 9) vs. 63.51 ± 13.73 %, (n = 5). Further, the suppression was ‘V’ shaped with suppression at SP dose of 2 µg/µl being significantly stronger from that observed with SP dose of 1 µg/µl and 7 µg/µl. The suppressive effects of SP dose of 1 µg/µl and 7 µg/µl were similar. The ‘control’ PS amplitudes were similar across all experimental groups (Groups, F_3, 22_ = 2.22, p > 0.10; one-way ANOVA with Newman-Keuls post-hoc test; data not shown).

Based on the preceding, we selected the SP dose of 2 µg/µl for experiments in awake animal, the dose of SP being functionally most efficacious among the different doses tested.

Effect of intraseptal microinjection of SP on reflexive behaviour to aversive stimulation (Fig. 2): Microinjection of SP into the MS region (Fig. 2A) of sham ligated animals decreased the PWT (Fig. 2B, left; Groups, F_5, 60_ = 9.90, p < 0.0001; one-way ANOVA followed by Newman Keuls post-hoc test). Likewise, the administration of the drug decreased the PWL (Fig. 2B, right; Groups, F_5, 60_ = 27.62, p < 0.0001). These effects are reminiscent of peripheral hypersensitivity (PH) observed on tissue injury, i.e. intraseptal SP evoked PH-like effects on reflexes. Interestingly, a small but significant decrease in PWL, but not PWT was observed with microinjection of SP into LS and with carbachol into the MS. This effect was however weaker compared to that seen with microinjection of SP in the MS (PWL: SP MS Sham vs. SP LS Sham vs. Carbachol MS Sham − 9.59 ± 1.84 vs. 13.80 ± 0.65 vs. 13.56 ± 0.77 s; Groups, F_2, 11_ = 18.52, p < 0.0004). The preceding is consistent with a stronger effect of microinjection of SP into MS.

The PH-like effects induced on intraseptal microinjection of SP in sham ligated animals was compared to PH evoked in another group of vehicle-treated animals following CCI. Behavioural observations were made from 15 min onwards after microinjection. The microinjections sites in both group of animals were in MS (data not shown). The ‘Baseline’ PWT and PWL were similar in the preceding two groups of animals. Similarly, the drop in value of PWT on SP microinjection and on microinjection of vehicle in CCI animals was similar. Thus, the PWT in animals on microinjection of SP in the MS region (28.75 ± 2.50, n = 4) was statistically not different from the PWT measured ipsilateral to CCI (23.33 ± 8.29, n = 9; p > 0.20, two tailed unpaired *t*-test). However, the PWL observed on microinjection of SP into the MS region (9.59 ± 1.84, n = 4) was significantly higher than that seen ipsilateral to CCI (4.67 ± 1.61, n = 9; p < 0.05, two tailed unpaired *t*-test), suggesting that CCI-induced thermal hypersensitivity is much more intense that SP-induced thermal hypersensitivity.

Effect of atropine on SP-induced changes in reflexes (data not shown): In a separate group of sham-ligated animals, atropine (5 mg/kg, i.p.) was administered prior to intraseptal microinjection of SP (data not shown). The intraseptal microinjections were all in the MS region (data not shown). The SP-induced responses of both the left and right hind paw in the experiment were similar and, thus, averaged as bilateral responses. The PWT observed on microinjection of SP in animals treated with atropine were of significantly higher value as compared to intraseptal microinjection of SP in the vehicle treated animals (data not shown; PWT, Groups x Timepoints, F_3, 27_ = 5.84, p < 0.004; two-way ANOVA followed by Bonferroni post-hoc test). Likewise, in case of the PWL (data not shown; PWL, Groups x Timepoints, F_3, 27_ = 27.78, p < 0.0001). The preceding findings suggest that atropine attenuated SP-induced PH-like effects.

To further assess the effect of atropine, we examine the effect of atropine on SP-induced hypersensitivity vis-à-vis ‘Baseline’ in the same group. Indeed, intraseptal SP failed to affect PWT in atropine treated animals compared to the corresponding ‘Baseline’ (data not shown; Groups, F_3, 15_ = 2.88, p > 0.07; one-way RM ANOVA with Newman-Keuls post-hoc test). Likewise, in case of the PWL (Groups, F_3, 15_ = 0.23, p > 0.80). Whereas a significant decrease was observed on microinjection of SP in vehicle treated animals (data not shown; PWT, Groups, F_3, 12_ = 90.00, p < 0.0001; PWL, Groups, F_3, 12_ = 30.87, p < 0.0001; one-way RM ANOVA with Newman-Keuls post-hoc test). The findings suggest that atropine blocked the ability of intraseptal SP to induce PH-like effects.

Effect of intraseptal SP in CPA (Fig. 3): These experiments investigated whether microinjection of SP *per se* into MS (Fig. 3A) was aversive to animals. Behavioural analysis indicated that microinjection of SP into the MS region of sham-ligated animals evoked a robust avoidance behaviour with the animal avoiding the preferred chamber that was paired with microinjection of SP into the MS region (Fig. 3B; Groups, F_2, 12_ = 44.78, p < 0.0001; one-way ANOVA followed by Newman-Keuls post-hoc test). Interestingly, a small, but significant effect was also seen on microinjection of SP into LS. There was no difference in the time spent by the different group of animals in the preferred and the non-preferred compartments during the two days of conditioning preceding the day of microinjection (Groups, F_5, 24_ = 0.388, p > 0.80; one-way ANOVA followed by Newman-Keuls post-hoc test; data not shown).

***Intraseptal L-733,060, an antagonist at NK1R, attenuated nociception in the CCI model***.

General: The preceding showed that intraseptal microinjection of SP, especially into MS, is aversive. Here we have investigated whether microinjection of L-733,060, an antagonist at NK1R, is antinociceptive in the CCI model of neuropathic pain. The effect of the antagonist was examined for an effect on both behavioural indices and cellular markers of nociception in mPFC and amygdala (Ariffin et al., 2018), regions implicated in nociception. It is interesting to note that the septal cholinergic neurons project to both mPFC (see ‘Introduction’) and the amygdala (Woolf and Butcher, 1982), besides the hippocampus. Furthermore, the hippocampal formation, the mPFC and the amygdala are functionally linked in modulation of affect-motivation (Beerens et al., 2021, Neugebauer, 2015). Thus, we reasoned that manipulating MS would affect nociceptive responses in these two regions in parallel with an effect on nociceptive behaviours. Additionally, we examined the effect of septal manipulations on nociception-related cellular changes in the spinal cord to investigate whether such manipulations evoke an effect with ramifications along the spinal-supraspinal neural axis (Ariffin et al., 2018).

The cellular markers included the following: (a) pERK in the mPFC region of the prelimbic/infralimbic (PrL/IL) regions, and the cingulate cortex (Cg) areas 1 and 2 (Cg1/Cg2), (b) pERK in basolateral amygdala (BLA), and the lateral (CeL) and capsular (CeC) regions of the central amygdala, and (c) pp38 and Iba1 in the dorsal horn of the spinal cord. pERK, besides reflecting cellular change to nociception, is also implicated in nociceptive affect-motivation especially in mPFC (Han et al., 2014). The spinal expression of Iba1 and the induction of pp38-ir are readouts of microglial activation. The spinal microglia are implicated in neuropathic nociception (Jin et al., 2003, Narita et al., 2006).

While, we have not examined the effect of intraseptal L-733,060 on cellular markers in hippocampus, we have previously reported that intraseptal L-733,060 attenuates electrophysiological markers of nociception in hippocampus in both anaesthetized and behaving rat in the formalin model of inflammatory pain (Ng et al., 2021).

Experimenters were blind to drug administered to animals.

Effect of intraseptal L-733,060 on PH (Fig. 4): Microinjection of L-733,060 or vehicle into the MS region or LS of sham ligated animals was without an effect on PWL and PWT of the ipsilateral paw (Groups x Timepoints, F_2, 24_ = 0.33 at least, p > 0.30 at least; two-way RM ANOVA with Bonferroni post-hoc test). Neither did microinjection affect PWT and PWL contralateral to sham ligation (Groups x Timepoints, F_2, 24_ = 0.46 at least, p > 0.30 at least; two-way RM ANOVA with Bonferroni post-hoc test; data not shown). The two groups of animals were thus combined to form the ‘Vehicle/L-733,060 MS/LS Sham’ group (n = 14) and their behavioural responses were compared with behavioural responses observed on microinjections in animals subject to CCI (Fig. 4A).

Two-way ANOVA followed by Bonferroni post-hoc test analysis showed that microinjection of L-733,060, but not vehicle, into the MS region or the LS (Fig. 4A) significantly reduced the CCI-induced PH in the ipsilateral paw (Fig. 4B; Ipsilateral PWT, Groups x Timepoints, F_6, 76_ = 16.35, p < 0.0001; Ipsilateral PWL, Groups x Timepoints, F_6, 76_ = 81.51, p < 0.0001). The antinociceptive effect was relatively more marked on PWT than on PWL, the former approaching the control values seen in Sham animals. Intraseptal microinjection of vehicle or L-733,060 in CCI animals did not affect PWT and PWL of the contralateral paw (Groups x Timepoints, F_6, 76_ = 0.51 at least, p > 0.50 at least; data not shown).

Effect of intraseptal L-733,060 on animal behaviour in CPP (Fig. 4C, D): Behavioural analysis indicated that microinjection of L-733,060 into the MS region or the LS of CCI-ligated animals (Fig. 4C) evoked a robust change in chamber preference. Thus, the animal spent more time in the in the non − preferred chamber paired with microinjection of L-733,060 into the septum (Fig. 4D; Groups, F_3, 23_ = 11.81, p < 0.0001; one-way ANOVA followed by Newman-Keuls post-hoc test). There was no difference in the time spent by the different group of animals in the preferred or the non-preferred compartments during the two days of conditioning preceding the day of microinjection (Groups, F_7, 46_ = 1.90, p > 0.09, one-way ANOVA with Newman-Keuls post-hoc test; data not shown).

Note that PH was observed in the ipsilateral paw of animals following CCI, but not sham surgery, when tested preceding the conditioning experiment (Time x Treatment, F_6, 100_ = 156.40 at least, p < 0.0001; two-way ANOVA followed by Bonferroni post-hoc test; data not shown).

Effect of intraseptal L-733,060 on peripheral stimulus-evoked cellular responses (Fig. 5, Fig. 6): The brain and spinal cord of animals used in behavioural tests for PH described above were prepared for immunohistochemistry after the last test of PWL. The immunohistochemical labelling was performed on alternate sections taken through the CNS regions of interest.

As previously described (Ariffin et al., 2018), the numbers of pERK-ir neurons were averaged bilaterally in the PrL/IL regions and Cg1/Cg2. This was so since the numbers of neurons on the side ipsilateral and contralateral to CCI in each of the above regions were similar. Likewise, in context of the BLA, CeL and CeC regions of the central amygdala. The numbers of pp38-ir were counted for the ipsilateral dorsal horn, while the relative density of Iba1-ir was calculated as ratio of densities of the ipsilateral to contralateral dorsal horn. These cellular markers were analysed in laminae I-IV (dorsal horn) of the lumbar spinal cord.

Overall, microinjection of L-733,060 into the MS region attenuated the expression of cellular markers in the forebrain (Fig. 5, Fig. 6). Further, the effect of drug administration into the MS region was more extensive compared to microinjections into LS. Thus, microinjection of L-733,060 into the MS region, but not LS of animals with CCI decreased pERK-ir in PrL/IL (Fig. 5, Fig. 6; Groups, F_4, 37_ = 28.38, p < 0.0001; one-way ANOVA followed by Newman Keuls post-hoc test), CeL (Fig. 5, Fig. 6; Groups, F_4, 37_ = 9.02, p < 0.001), and CeC (Fig. 5, Fig. 6; Groups, F_4, 37_ = 20.78, p < 0.001). In contrast, the expression of pERK-ir in Cg was reduced by microinjection of L-733,060 into both the MS region and the LS of CCI-ligated animals (Groups, F_4, 37_ = 37.12, p < 0.0001; Fig. 5, Fig. 6).

The cellular effects of L-733,060 were forebrain specific since microinjection of the antagonist into the MS region did not affect the expression of pp38-ir (Groups, F_2, 11_ = 22.30, p < 0.0002; Fig. 5, Fig. 6) and Iba1-ir (p < 0.002; Kruskal-Wallis test followed by Dunn’s post-hoc test; Fig. 5, Fig. 6) in the spinal cord of CCI animals. We did not investigate the effect of microinjection into the LS.

Interestingly, while an increase of pERK-ir was observed in BLA (p < 0.002; Kruskal-Wallis test followed by Dunn’s post-hoc test; data not shown) neither microinjection of L-733,060 into the MS region or the LS of CCI animals had an effect.

***Decreasing septo-hippocampal cholinergic function strongly attenuates CCI-induced nociception***

General: The preceding series of experiments suggests that activation of intraseptal NK1R, which are localized on septal cholinergic neurons in the MS region, evokes an atropine sensitive nociception in naïve animals, which is consistent with a role of septal cholinergic neurons in mediating nociception. Further, the septal NK1R mediate, at least in part, nociception in the CCI model. To extend the above observations, here we test whether septo-dorsal hippocampal cholinergic neurotransmission underpin, at least in part, nociception in the CCI model. Notably, the hippocampus receives cholinergic inputs exclusively from the MS region.

Effect of intraseptal 192 IgG-SAP on the numbers of septal cholinergic neurons (data not shown): As previously (Ariffin et al., 2013, Zheng and Khanna, 2001), pre-treatment with 192 IgG-SAP evoked a robust loss of ChAT-ir neurons, putative cholinergic neurons, in the MS region. The loss was observed in the different areas of the MS region. These include the MS nucleus, vertical diagonal band of Broca and horizontal diagonal band of Broca (Groups, F_3, 40_ = 66.46 at least, p < 0.0001, one-way ANOVA followed by Newman-Keul’s post-hoc test). The loss was selective since the numbers of PV-ir putative GABAergic neurons, were not affected by 192 IgG-SAP (Groups, F_3, 40_ = 1.95 at least, p > 0.0.05).

Effect of intraseptal 192 IgG-SAP on PH (data not shown): The animals were subject to behavioural evaluation at different stages of the experiment, i.e., ‘Baseline’ (before microinjection), ‘Lesion’, and ‘Ligation’. The ‘Lesion’ PWT and PWL were measured two weeks after intraseptal microinjection of 192 IgG-SAP or Vehicle. Following the measurements of PWT and PWL, the animals were subjected to CCI. Post-ligation, the test for PWT and PWL were performed on day 5 and 7, respectively (‘Ligation’).

Lesion of the septal cholinergic neurons with 192 IgG-SAP strongly attenuated the development of PH in the ipsilateral paw of CCI-ligated animals (PWT, Groups x Timepoints, F_6, 80_ = 44.42, p < 0.0001; PWL, Groups x Timepoints, F_6, 80_ = 33.28, p < 0.0001; two-way ANOVA followed by Bonferroni post-hoc test). Indeed, the PWT and the PWL of the injured paw in 192 IgG-SAP pre-treated animals was similar to the corresponding values in vehicle or 192 IgG-SAP pre-treated sham animals. The PWT and PWL of 192 IgG − SAP pre − treated sham animals were not different from the corresponding values from sham animals pre-treated with vehicle, suggesting that intraseptal 192 IgG-SAP *per se* did not influence physiological reflexes.

Effect of intraseptal 192 IgG-SAP on cellular responses in CCI (Fig. 7A, B): In the animals tested above, the loss of cholinergic neurons with 192 IgG-SAP pre-treatments attenuated and or prevented the induction of cellular markers of nociception in forebrain and spinal cord. Thus, the lesion prevented an increase in the number of pERK-ir neurons in PrL/IL and Cg1/Cg2 regions of CCI animals that was otherwise observed in control animals (Fig. 7A; PrL/IL, Groups, F_3, 24_ = 4.63, p < 0.02; Cg1/Cg2, F_3, 24_ = 24.27, p < 0.0001; one-way ANOVA followed by Newman-Keuls post-hoc test). Similarly, the lesion prevented the induction of pp38-ir in CCI animals (Fig. 7B; Groups, F_3, 15_ = 41.26, p < 0.0001; one-way ANOVA followed by Newman-Keuls post-hoc test). However, Iba1-ir was induced, although at a level lower than in control animals (Fig. 7B; Groups, F_3, 15_ = 32.61, p < 0.0001).

Effect of bilateral intra-cingulate antagonists on CCI-induced PH (Fig. 8): The two antagonists, namely atropine and mecamylamine, were microinjected bilaterally into the Cg1 region of the mPFC (Fig. 8A). The two antagonists were microinjected in separate experiments.

The effect of the microinjection of atropine (‘Atro Cg1 CCI’, n = 7) or mecamylamine (‘Mec Cg1 CCI’, n = 7) in CCI animals was compared with a ‘Veh/Atro/Mec Cg1 Sham’ group (n = 17). Notably, the ‘Veh/Atro/Mec Cg1 Sham’ group included 3 different groups, namely sham ligated animals microinjected into Cg1 with (a) vehicle (n = 5), (b) atropine (n = 8) or (c) mecamylamine (n = 4). The PWTs or the PWLs in these three groups was very similar (Groups x Timepoints, F_4, 28_ = 0.68 at least, p > 0.20 at least; two-way RM ANOVA followed by Bonferroni post-hoc test). Thus, the three groups were combined to form the ‘Veh/Atro/Mec Cg1 Sham’ group (n = 17).

Two-way ANOVA followed by Bonferroni post-hoc test showed that microinjection of atropine or mecamylamine reversed CCI-induced PH in the ipsilateral paw toward the values observed in ‘Veh/Atro/Mec Cg1 Sham’ group (Fig. 8B; PWT, Groups x Timepoints, F_6, 62_ = 55.05, p < 0.0001; PWL, Groups x Timepoints, F_6, 62_ = 29.90, p < 0.0001). Note that the PWT and PWL in ‘Veh/Atro/Mec Cg1 Sham’ were very similar at ‘Baseline’, ‘Ligation’ (i.e., sham-ligation) and ‘Microinjection’ (Fig. 8B). Additionally, the antagonists did not affect contralateral PWT and PWL in the ‘Atropine Cg1 CCI’ and ‘Mecamylamine Cg1 CCI’ groups, the values being comparable to the corresponding values from the ‘Veh/Atro/Mec Cg1 Sham’ groups (Groups x Timepoints, F_6, 62_ = 0.18 at least, p > 0.80 at least; two-way RM ANOVA followed by Bonferroni post-hoc test; data not shown). This indicates that the intra-cingulate microinjection of the antagonists did not affect physiological responses.

Effect of bilateral dH microinjection of antagonists on CCI-induced PH (Fig. 9): The microinjection sites in dH are illustrated in Fig. 9A. In animals that underwent CCI, the combination of bilateral microinjection sites in dH are as follows: (a) bilateral microinjection of vehicle (‘Veh dH CCI’ group, n = 6; Fig. 9A): CA1 on both sides or CA1/CA1 (n = 3), CA3/CA3 (n = 1), DG/DG (n = 1) and CA1/CA3 (n = 1), (b) bilateral microinjection of atropine (‘Atro dH CCI’, n = 9; Fig. 9A): CA1/CA1 (n = 2), CA3/CA3 (n = 2), CA1/CA3 (n = 3), and CA3/DG (n = 2), and (c) bilateral microinjection of mecamylamine (‘Mec dH CCI’ group, n = 8; Fig. 9A): CA1/CA1 (n = 1), CA3/CA3 (n = 1), DG/DG (n = 1), CA1/CA3 (n = 1), CA1/DG (n = 2), CA3/DG (n = 1), and CA1/CA3 (n = 1).

The sham-ligated group of animals were microinjected bilaterally into dH with either (a) vehicle (‘Veh’, n = 4), (b) atropine (‘Atro’, n = 4) or (c) mecamylamine (‘Mec’, n = 5). The PWTs or the PWLs in these three groups were very similar (Groups x Timepoints, F_4, 20_ = 0.00 at least, p > 0.09 at least; two-way RM ANOVA followed by Bonferroni post-hoc test). Thus, the three groups were combined to form the ‘Veh/Atro/Mec dH Sham’ group. The distribution of microinjection sites in dH in the ‘Veh/Atro/Mec dH Sham’ group (n = 13; Fig. 9A) were as follows: CA1/CA1 (n = 3), DG/DG (n = 3), CA3/CA1 (n = 3), CA1/DG (n = 3), and CA3/DG (n = 1).

Two-way ANOVA followed by Bonferroni post-hoc test showed that microinjection of atropine or mecamylamine reversed CCI-induced PH of the ipsilateral paw toward the values observed in ‘Veh/Atro/Mec dH Sham’ group (Fig. 9B; PWT, Groups x Timepoints, F_6, 64_ = 22.59, p < 0.0001; PWL, Groups x Timepoints, F_6, 64_ = 15.78, p < 0.0001). Note that the PWT and PWL in ‘Veh/Atro/Mec dH Sham’ were very similar at ‘Baseline’, ‘Ligation’ (sham-ligation) and ‘Microinjection’ (Fig. 9B). Additionally, the antagonists did not affect contralateral PWT and PWL in the ‘Atro dH CCI’ and ‘Mec dH CCI’ groups, the values being comparable to the corresponding values from the ‘Veh/Atro/Mec dH Sham’ groups (Groups x Timepoints, F_6, 64_ = 0.89 at least, p > 0.30 at least; two-way RM ANOVA followed by Bonferroni post-hoc test; data not shown).

Effect of unilateral dH microinjection of cholinergic receptor antagonists on CCI-induced PH (Fig. 9): We also examined whether the effect of antagonists on microinjection into hippocampus was lateralized. In this context, we repeated the above experiment in a different set of animals with microinjections of either atropine, mecamylamine or vehicle into either the left (contralateral to CCI) or the right (ipsilateral to CCI) dH. The microinjection sites are illustrated in Fig. 9C. Of the 49 sites, 40 were localised in the CA1 region, 1 was in the field CA3, and 8 were localized to the DG (Fig. 9C). In addition, in 6 animals, atropine (n = 3) or mecamylamine (n = 3) was microinjected dorsal to hippocampus (Fig. 9C). These animals comprised the ‘Out of Site’ (OOS, n = 6) group.

In sham-ligated animals, the PWT and the PWL of paws ipsilateral or contralateral to the sham surgery were unaffected on microinjection into the dH (Vehicle: contralateral dH, n = 5, ipsilateral dH, n = 3; Atropine: contralateral dH, n = 4; Mecamylamine contralateral dH, n = 3; Groups x Timepoints, F_6, 22_ = 0.52 at least, p > 0.30 at least; two-way RM ANOVA followed by Bonferroni post-hoc test; data not shown). These groups were combined to form the ‘Veh/Atr/Mec uni-dH Sham’ group (n = 15). Note that the lack of effect of antagonists on PWL and PWT in sham animals is consistent with the lack of effect of bilateral microinjections of the antagonist in sham animals as described above.

Similarly, vehicle administration into the ipsilateral (n = 5) or contralateral (n = 5) dH of CCI ligated animals did not affect the PWT or PWL of the ipsilateral paw (Groups x Timepoints, F_2, 16_ = 1.26 at least, p > 0.10 at least; two-way RM ANOVA followed by Bonferroni post-hoc test; data not shown), and as such the two groups were combined to form the ‘Veh uni-dH CCI’ group (n = 10). The PWT and the PWL of the paw contralateral to CCI was also not affected by microinjection of vehicle in the preceding group (Groups x Timepoints, F_2, 16_ = 0.03 at least, p > 0.05 at least; two-way RM ANOVA followed by Bonferroni post-hoc test; data not shown).

Two-way ANOVA followed by Bonferroni post-hoc test showed that microinjection of the cholinergic antagonists into dH, but not OOS, attenuated CCI-induced PH. The effect of intra-hippocampal microinjections was observed regardless of whether the drugs were microinjected into dH ipsilateral or contralateral to CCI. Both PWT (Fig. 9D; Groups x Timepoints, F_12, 96_ = 22.90, p < 0.0001) and PWL (Fig. 9D; Groups x Timepoints, F_12, 96_ = 38.99, p < 0.0001) were attenuated. Microinjections of the antagonists into dH had no effect on the contralateral paw (Groups x Timepoints, F_12, 96_ = 1.27 at least, p > 0.10 at least; data not shown).

Effect of bilateral microinjection of cholinergic receptor antagonists on cellular changes in the CCI-model: Only the effects of bilateral intra-hippocampal microinjection of atropine or mecamylamine were investigated. The brain and spinal cord of animals used in the behavioural tests above (Fig. 9B) were prepared for immunohistochemistry after the last test of PWL. The immunohistochemical labelling was performed for c-Fos-ir on alternate sections taken through the CNS regions of interest. cFos was particularly used as a marker for this part of the study since it has been shown before that noxious stimuli evoke a decrease in c-Fos in dorsal CA1, suggesting an overall decrease in activity of hippocampal neurons (Khanna et al., 2004). This is consistent with noxious stimulus-induced pyramidal cell suppression observed in electrophysiological studies (Khanna, 1997). As previously (Ariffin et al., 2018, Khanna et al., 2004), cFos-ir was averaged bilaterally for each section and averaged again over the number of sections.

Overall, the acute effect intra-hippocampal cholinergic antagonists mimicked the findings seen on lesion of septal cholinergic neurons. Thus, compared to microinjection of vehicle, microinjection of cholinergic receptor antagonists into dH of CCI animals significantly attenuated the number of cFos-ir neurons in the PrL/IL and Cg1/Cg2 regions in animals (Fig. 10A; PrL/IL, Groups, F_3, 20_ = 10.44, p < 0.0003; Cg1/Cg2, Groups, F_3, 20_ = 14.28, p < 0.0001; one-way ANOVA followed by Newman-Keuls post-hoc test).

Spinal levels of Iba1-and pp38-ir were also attenuated on microinjection of the cholinergic antagonists into dH (Fig. 10B; Iba1, Groups, F_3, 25_ = 22.51, p < 0.0001; Fig. 10C; pp38, Groups, F_3, 25_ = 30.10, p < 0.0001; one-way ANOVA followed by Newman-Keuls post-hoc test).

In context of dorsal CA1, the numbers of c-Fos-ir cell in dH CA1 of CCI animals were similar for groups microinjected with atropine (82.32 ± 43.74, n = 4) or mecamylamine (91.15 ± 13.79, n = 4). Thus, two groups were combined. The number of c-Fos-ir neurons per section of sham animals microinjected with either vehicle, atropine or mecamylamine were also similar. Thus, these animals were also grouped together (71.55 ± 16.63, n = 6). The number of c-Fos-ir neurons per section of sham animals (n = 6) vs. CCI animals’ microinjected with vehicle into the MS region (n = 4) vs CCI animals’ microinjected with either atropine or mecamylamine (n = 8) were as follows: 71.55 ± 16.63 vs. 39.47 ± 12.45 vs. 86.74 ± 30.39. One-way ANOVA followed by post-hoc Newman-Keuls test showed an overall significant effect of treatment, with c-Fos in CCI animals’ microinjected with vehicle being significantly lower as compared to c-Fos in CCI animals’ microinjected with either atropine or mecamylamine (Fig. 11; Groups, F_2, 15_ = 5.378, p < 0.05). The c-Fos in CCI animals microinjected with vehicle was also tended to be lower (by ∼ 45 %) as compared to sham animals, although not significantly so. The c-Fos in sham animals were no different from c-Fos in CCI animals microinjected with either atropine or mecamylamine. The findings suggest that the test of PH evoked a decrease in c-Fos in dH CA1 which is prevented by intra-hippocampal microinjection of cholinergic antagonists.

Analysis of c-Fos −ir in the ventral hippocampal CA1 fields (vH) did not reveal any significant difference between the groups (Groups, F_2, 15_ = 2.21, p > 0.10; One-way ANOVA followed by post-hoc Newman-Keuls test). The average number of cFos-ir neurons per section of sham animals (n = 6) vs. CCI animals’ microinjected with vehicle into the MS region (n = 4) vs CCI animals’ microinjected with either atropine or mecamylamine (n = 8) were as follow: 272.21 ± 103.05 vs. 218.94 ± 56.79 vs. 195.40 ± 31.00.

## Discussion

The present study has led to two main novel findings. One, septo-dH cholinergic transmission mediates experimental neuropathic pain. Thus, on one hand, intraseptal SP, a NKR agonist, evoked PH-like response in rat that was antagonized by atropine, which is consistent with the notion that intraseptal SP excites septal cholinergic neuron (Morozova et al., 2008). Indeed, atropine also antagonizes intraseptal SP-induced suppression of CA1 pyramidal cell excitability (Ng et al., 2021). On the other hand, selective destruction of septal cholinergic neurons in rat with the immunotoxin, 192 IgG-SAP, prevented the development of experimental neuropathic pain and the induction of nociceptive markers in the spinal cord and forebrain.

Moreover, microinjection of the cholinergic antagonists, atropine or mecamylamine, into the dH of rat following CCI evoked a therapeutic-like antinociceptive effect and reversed PH. In parallel, the intra-hippocampal cholinergic antagonists blocked a nociception-induced changes in c-Fos in the forebrain and the induction of nociceptive markers in spinal cord. Interestingly, the antinociceptive effect of the cholinergic antagonists was distributed throughout the dH. Thus, anti-nociception was evoked both on bilateral or unilateral microinjection of the cholinergic receptor antagonists, atropine or mecamylamine, into the different regions of the dH. The distributed effect of cholinergic antagonists parallels the observation that the hippocampal nociceptive processing is distributed (Khanna et al., 2004, Shao et al., 2023, Wang et al., 2024). Accordingly, therefore, interrupting the distributed processing at any point of the network was antinociceptive.

Interestingly, the microinjection of the cholinergic antagonists attenuated the decrease in the induction of c-Fos in dH CA1 in the CCI model suggesting that the antagonists attenuated the noxious stimuli-induced suppression of neuronal excitability in the region. In this regard, we have proposed a model that envisages an inhibitory modulation of CA1 pyramidal cells by septal cholinergic neurons during nociceptive processing in the region. In this model, the septo-hippocampal cholinergic neurons acting through hippocampal muscarinic receptors evoke an inhibition of pyramidal cells discharge in dH field CA1 via excitation of the interneuronal network that is accompanied by a muscarinic receptor-mediated suppression of intrinsic excitatory synaptic input connecting DG-CA3-CA1 (Hasselmo and McGaughy, 2004, Khanna, 1997, Khanna and Zheng, 1999, Lovett-Barron et al., 2014, Zheng and Khanna, 1999, Zheng and Khanna, 2001, Zheng and Khanna, 2008). Pertinent here, the nicotinic receptor mechanism also mediates excitation of hippocampal inhibitory interneuron in hippocampus (Haam and Yakel, 2017).

Collectively, the preceding findings suggests that the septo-hippocampal cholinergic transmission suppresses hippocampal excitatory neurons, especially in CA1, through muscarinic and nicotinic receptor mechanisms during persistent nociception. Indeed, persistent nociception is linked to a decrease in neural excitability in hippocampus (Jiang et al., 2018, Klamt and Prado, 1991, Mendes and Menescal-de-Oliveira, 2008, Shao et al., 2023, Wang et al., 2024, Wei et al., 2021). The circuit mechanisms that underpin the cholinergic mediation of nociception via dH is unclear. Notably, however, the dH, especially neurons in CA1 project to dorsal subiculum that is considered a major output from the hippocampal formation. In turn, the dorsal subiculum projects to the mPFC, particularly the anterior Cg (Beerens et al., 2021, Rizzi-Wise and Wang, 2021). Here it is notable that a direct septal-Cg cholinergic projection is implicated in mediation of persistent nociception by exciting Cg neurons (Jiang et al., 2018). Perhaps, the indirect septal cholinergic-dH-dorsal subiculum-Cg projection reinforces the effect of the direct projection since we show that intra-hippocampal cholinergic antagonists also attenuate the induction of c-Fos in mPFC in the CCI model. The induction of c-Fos in Cg and PrL regions and, in general, an increase in excitability of pyramidal neurons in the mPFC, especially Cg and to some extent in PrL is linked to mediation of persistent nociception (Blom et al., 2014, Cao et al., 2022, Chen et al., 2014, Chen et al., 2018; Chung et al., 2017, Zhang et al., 2015; Zhuo, 2013).

Consistent with a role of Cg cholinergic mechanisms in nociception, we observed that microinjection of atropine or mecamylamine into the Cg attenuated PH in the CCI model. However, how the cholinergic transmission affects nociception through Cg is not clear. The cholinergic transmission in mPFC, especially transmission at muscarinic receptors both excites and inhibits pyramidal cells, either directly or indirectly (Obermayer et al., 2017, Hedrick and Waters, 2015; Zhang and Philippe, 2010). An impaired cholinergic mediated excitation of pyramidal cells at M1 muscarinic receptors in PrL and an attenuation of M1 muscarinic receptor-mediated inhibition of pyramidal cells in Cg facilitates nociception (Jefferson et al., 2023, Koga et al., 2017, Koga et al., 2019; Radzicki et al., 2017). The anti-nociceptive effects of intra-cingulate atropine in the present study is unlikely to be associated with antagonism of cholinergic transmission at M1 receptor, as such antagonism will lead to enhanced nociception. However, the drug is a broad-spectrum antagonist at muscarinic receptors and might non-selectively antagonize the varied effects of cholinergic transmission at different muscarinic receptors that might, perhaps, include a muscarinic-mediated excitation of Cg pyramidal cells in nociception. Notably, excitation of Cg pyramidal cells is linked to nociception (see above).

Two, septal NKRs selectively mediate experimental neuropathic pain. Indeed, the NKR agonist, SP, was relatively selective in mediation of peripheral hypersensitivity vis-à-vis intraseptal microinjection of carbachol. The relative insensitivity of carbachol was observed even though the drug at the dose used evoked a broad-based activation of the MS region marked by a similar level of suppression of hippocampal CA1 PS compared to SP and the generation of robust hippocampal theta wave activity (Ng et al., 2021). This suggests that activation of NK1R in the MS, as opposed to wider MS activation, selectively mediate an aversive valence. The NK1R mediation of nociception is, presumably, in part due to a selective activation of septal cholinergic neurons that are implicated in nociception. On the other hand, the more broad-based activation with intraseptal carbachol might weigh in favour of more complex behaviours than reflexive nociceptive changes. As noted above, carbachol evokes septo-hippocampal theta that is associated with animal voluntary behaviour (Bender et al., 2015, Fuhrmann et al., 2015, Lawson and Bland, 1993, Monmaur et al., 1997). Indeed, in context of nociception, a block of theta rhythmogenesis with intraseptal bicuculline, a GABA_A_ receptor antagonist, dramatically increases formalin-induced ambulation, though, nociceptive licking and flinching are not affected (Ang et al., 2015). Interestingly, septal GABAergic and glutamatergic, but not cholinergic neurons are implicated in theta rhythmogenesis, and modulate ongoing animal explorations (Bender et al., 2015, Fuhrmann et al., 2015; Vandecasteele et al., 2014).

Moreover, other data from the study strengthens the idea that activation of NK1R mediates aversive valence. Thus, intraseptal SP also evoked aversion marked by CPA, whereas, conversely, the NK1R antagonist, L-733,060, evoked antinociception in the CCI model without eliciting a general decrease in adaptive behaviour. In this regard, intraseptal L-733060, partially reduced PH without affecting physiological reflexes and facilitated CPP, reflecting learned/adaptive behaviour (present study). Earlier we have shown that the drug has no effect on animal ambulatory behaviour during nociception and spares the exploration- and direct septal stimulation-induced hippocampal theta in behaving animal (Ng et al., 2021). The drug also antagonized nociception-induced neural changes, including theta power in the hippocampus (Ng et al., 2021). The septal NK1R mediation of nociception might be part of the selective role of NK1R in nociceptive processing along the spinal-septal-hippocampal relay on tissue damage. Notably, the MS region receives spinal projections from NK-1 receptor positive neurons (Li et al., 1997), while the septal neurons, especially the cholinergic neurons that exclusively express NKRs in MS region project to the hippocampus. NK1R in spinal cord mediate nociception (Zieglgänsberger, 2019), while NK1R levels in hippocampus are also affected with nociception (Duric and McCarson, 2005). In the MS region, the NK1R might facilitate an aversive imprint in a cooperation with the ubiquitous excitatory transmitter glutamate which has also been implicated in septal mediation of nociception (Ariffin et al., 2018, Ibrahim et al., 2021).

Strikingly, the effect of intraseptal SP on PWT and PWL in naïve animals was relatively mild as compared to CCI, even though SP was microinjected at a maximal dose in mediating a cholinergic septo-hippocampal response. This contrast with the robust effect of L-733,060 in inducing CPP. In this regard, the degree of CPP evoked on intraseptal L-733,060 is comparable to that evoked with intraseptal muscimol, a GABA mimetic (Ariffin et al., 2018). In contrast to L-733,060, muscimol, however, also blocked PH, and forebrain and spinal nociceptive cellular processing. Whereas intraseptal L-733,060 attenuate forebrain nociceptive processing while sparing spinal nociception. The similarity of effect of L-733,060 compared with muscimol on CPP suggests that septal NK1R preferentially influences nociceptive affect-motivation via modulations of forebrain regions while inducing a minimal change in PH and spinal nociceptive processing. Indeed, it is notable that intraseptal L-733,060 attenuated the expression of pERK in mFC. pERK in mPFC, especially in the Cg is associated with stimuli-induced aversion, a measure of affect, in the CCI model in the male rat (Han et al., 2014). The preferential anti-aversive effect at NK1Rs as compared to the robust effects of intra-hippocampal cholinergic antagonism on nociceptive transmission might be a functional reflection of a more limited excitation of septal cholinergic neurons through NKR due to internalization of these receptor on the binding of neurokinins (Jensen et al., 2017). Perhaps, reflecting such receptor dynamics, we find that the dose–response of intraseptal SP is a V-shape with lowering of functional effects at a higher dose. Although untested, perhaps, the intraseptal connectivity also limits the response to NK1R activation. Interestingly, the septal cholinergic neurons are interconnected with local non-cholinergic neurons, including inhibitory GABAergic neurons (see Ng et al., 2021). Potentially such interlinks, involving feedback inhibition by GABAergic neurons on excitation by cholinergic neurons, might functionally limit the strength of excitation of cholinergic neurons, perhaps being more apparent at the higher dose of SP.

Alternately, or conjointly, a potential countervailing effect of intraseptal SP-induced cholinergic activation, especially to vH, might limit the pro-nociceptive effects of intraseptal SP. In this regard, excitation of septo-vH cholinergic neurons is antinociceptive (Jiang et al., 2018). Although we have not examined the effect of intraseptal SP on septo-vH cholinergic neurons, it is notable that almost all (more than 80 %) cholinergic neurons in the MS region express NKRs that, conversely, are expressed exclusively in cholinergic neurons of the MS region (Chen et al., 2001). Furthermore, the bulk of septo-hippocampal cholinergic are excited by agonists at NKR, including SP that excites 95 % of the large population of neurons tested (Morozova et al., 2008). Likewise, for agonists at other NKRs (Morozova et al., 2008). In parallel, intraseptal microinjection of agonists at NKRs evokes a release of acetylcholine in temporal region of hippocampus that correspond to vH (Schäble et al., 2012). This raises the possibility that intraseptal SP excites the septo-vH cholinergic neurons.

The effect of microinjection of L-733,060 into the MS region on PH and CPP in CCI animals was co-extensive with the adjacent LS. The microinjection of SP into LS was also aversive, though less efficacious than the microinjections into MS region. The observed effects on microinjection into LS are unlikely to reflect an effect due to diffusion of the drug into the MS. In this context, we have shown previously that microinjection of SP into LS did not influence hippocampal neural activity, unlike that seen-on microinjection into the MS region (Ng et al., 2021). Likewise, L-733,060 did not affect noxious stimulus-induced hippocampal neural changes in behaving animal on microinjection into LS but attenuated the neural responses on microinjection into MS region (Ng et al., 2021).

Indeed, in line with above, the cellular effects of microinjection of L-733,060 into MS were much more widespread than those seen on microinjecting the agent into LS, although the behavioural effect were similar. This, in turn, suggests that the cellular changes observed on microinjection of L-733,060 into the MS region are not passive and secondary to behavioural change but, rather, reflect an active modulation of nociceptive activity in the forebrain. Furthermore, the dissociation in the effects of L-733,060 microinjected into MS vs. LS suggests that the NKR mechanisms in the MS and the LS regions mediate effects via separate mechanisms. For example, the NKR mechanism in MS likely integrate the cellular responses in the hippocampal and cingulate networks via the septal cholinergic neurons (present study; Jiang et al., 2018, Ng et al., 2021), while LS is reported to modulate nociception partly through the hypothalamic region (Li et al., 2023, Wang et al., 2023).

## Conclusion

In conclusion, the development and maintenance of neuropathic nociception is facilitated by septal NKR-cholinergic neurons involving, in part, the dH. The transmission at septal NKR is relatively selective for persistent nociception and modulates the affective component of pain. The preceding findings, seen in juxtaposition with published data, suggests that the (a) septal cholinergic neurons are a key mediator of aversion through diverse projections, including the septo-cingulate (Jiang et al., 2018), septo-habenular (Mu et al., 2022) and septo-dH (present study). Contrarily, the septo-vH cholinergic neurons that, unlike septo-dH cholinergic neurons, contain calbinding-D28K protein, is part of a separate stream which, together with cholinergic neurons of basal nucleus of Meynert and pedunculopontine tegmentum, evoke antinociception on activation (Jiang et al., 2018, Oswald et al., |2022, Sullere et al., 2023), and (b) anti-muscarinic and anti-NKRs drugs may potentially reinforce the effects of each other on pain, at least partly, by an effect on cholinergic transmission. Interestingly, recent evidence suggests that anti-muscarinic drugs also have a peripheral effect that facilitate antinociception and reverse or prevent indices of peripheral neuropathy, leading to a suggestion that anti-muscarinic can potentially be re-purposed for modulation of changes with neuropathy (Calcutt et al., 2017). Additionally, both experimental and clinical evidence suggests that NKR antagonists, including NK1R antagonist, are effective in ameliorating stress-induced aversive disorders (Ebner et al., 2009; Rupniak and Kramer, 2017). Collectively, the preceding suggests a potential strategy of modulating pain by combining anticholinergics and anti-NKRs that reinforce each through related or independent actions, perhaps leading to a greater efficacy and a wider therapeutic window.

## Credit authorship contribution statement

**Mohammed Zacky Ariffin:** Writing – original draft, Investigation, Formal analysis, Conceptualization. **Si Yun Ng:** Writing – review & editing, Writing – original draft, Investigation, Formal analysis, Conceptualization. **Hamzah Nadia:** Investigation, Formal analysis. **Darrel Koh:** Investigation, Formal analysis. **Natasha Loh:** Investigation, Formal analysis. **Naomi Michiko:** Investigation, Formal analysis. **Sanjay Khanna:** Writing – review & editing, Supervision, Funding acquisition, Conceptualization.

## Declaration of competing interest

The authors declare that they have no known competing financial interests or personal relationships that could have appeared to influence the work reported in this paper.

## Data Availability

Data will be made available on request.
